# System-level network analysis of nitrogen starvation and recovery in *Chlamydomonas reinhardtii* reveals potential new targets for increased lipid accumulation

**DOI:** 10.1186/s13068-014-0171-1

**Published:** 2014-12-24

**Authors:** Luis Valledor, Takeshi Furuhashi, Luis Recuenco-Muñoz, Stefanie Wienkoop, Wolfram Weckwerth

**Affiliations:** Department of Ecogenomics and Systems Biology, Faculty of Life Sciences, University of Vienna, Althanstrasse 14, A-1090 Vienna, Austria; Cyanoteam, Global Change Research Center-Czechglobe, Academy of Sciences of the Czech Republic, Belidla 4, 603 00 Brno, Czech Republic; Present address: Plant Physiology, University of Oviedo, Catedrático Rodrígo Uría s/n, E-33006 Oviedo, Spain

## Abstract

**Background:**

Nitrogen starvation is known to cause drastic alterations in physiology and metabolism leading to the accumulation of lipid bodies in many microalgae, and it thus presents an important alternative for biofuel production. However, despite the importance of this process, the molecular mechanisms that mediate the metabolic remodeling induced by N starvation and especially by stress recovery are still poorly understood, and new candidates for bioengineering are needed to make this process useful for biofuel production.

**Results:**

We have studied the molecular changes involved in the adaptive mechanisms to N starvation and full recovery of the vegetative cells in the microalga *Chlamydomonas reinhardtii* during a four-day time course.

High throughput mass spectrometry was employed to integrate the proteome and the metabolome with physiological changes. N starvation led to an accumulation of oil bodies and reduced Fv/Fm.. Distinct enzymes potentially participating in the carbon-concentrating mechanism (CAH7, CAH8, PEPC1) are strongly accumulated. The membrane composition is changed, as indicated by quantitative lipid profiles. A reprogramming of protein biosynthesis was observed by increased levels of cytosolic ribosomes, while chloroplastidic were dramatically reduced. Readdition of N led to, the identification of early responsive proteins mediating stress recovery, indicating their key role in regaining and sustaining normal vegetative growth.

Analysis of the data with multivariate correlation analysis, Granger causality, and sparse partial least square (sPLS) provided a functional network perspective of the molecular processes. Cell growth and N metabolism were clearly linked by the branched chain amino acids, suggesting an important role in this stress. Lipid accumulation was also tightly correlated to the COP II protein, involved in vesicle and lysosome coating, and a major lipid droplet protein. This protein, together with other key proteins mediating signal transduction and adaption (BRI1, snRKs), constitute a series of new metabolic and regulatory targets.

**Conclusions:**

This work not only provides new insights and corrects previous models by analyzing a complex dataset, but also increases our biochemical understanding of the adaptive mechanisms to N starvation in *Chlamydomonas*, pointing to new bioengineering targets for increased lipid accumulation, a key step for a sustainable and profitable microalgae-based biofuel production.

**Electronic supplementary material:**

The online version of this article (doi:10.1186/s13068-014-0171-1) contains supplementary material, which is available to authorized users.

## Background

The need for an alternative to fossil fuels, with rising prices and declining reserves, has led to renewed interest in microalgae as a potential source for biofuel production [1-3]. Nutrient availability, temperature, and light intensity are the major environmental determinants of algal growth, reproduction, and morphology, including the accumulation of lipids in the form of triacylglycerols (TAGs) [4-6]. Photoautotrophically grown algae offer better solar energy conversion efficiency and a range of technical and ethical advances compared to traditional oil crops [7-9]. Considering their increasing importance as bioproducers and the need to achieve an optimized balance between lipid production and growth, thorough analyses of the underlying molecular mechanisms that mediate stress-induced accumulation of lipids in microalgae are necessary. Nevertheless, these analyses are still in a very early stage [10].

Growth-inhibiting conditions and an imbalance between carbon and some macro- and micro- elements such as Fe, S, Zn, or N [11] lead to metabolic rearrangements modulating cell division, morphology, and photosynthetic capacity [12] and the accumulation of starch [13] and/or lipids [14] to protect cellular structures and increase the microalgae survival probability under adverse circumstances. The accumulation of lipid bodies in *Chlamydomonas reinhardtii* under N deficiency has been recently documented in detail [4,14,15], establishing a well-known environment in which changes in morphology and some key genes are defined.

These studies together with the availability of a sequenced genome [16], proteomics and metabolomics protocols and databases [17-27], pathway annotations [28-30], and a wide range of molecular biology [31] and transcriptomics tools [32] make *C. reinhardtii* the premier molecular model for research in microalgae.

The employment of recent advances in high throughput profiling methodologies has allowed the system-level characterization of *C. reinhardtii* at transcriptomic [11,15,33], proteomic, and metabolomic levels [19,21,34]. In the present study, we have added a further layer of investigation, in particular distinguishing short- and long-term adaptive mechanisms and the recovery phase of the cells from N starvation to normal vegetative growth. In contrast to previous studies on differential gene expression [33,35] or metabolomics analyses [34], we have studied N starvation and the following recovery process after N readdition during a four-day experiment. Using classical physiological measurements, mass spectrometry for quantitative proteomics (GeLC-LTQ-Orbitrap-MS) and metabolomic (GC-MS) changes, and mining available datasets we depicted the responses of *C. reinhardtii* to available N, showing the dynamic behavior of the biochemical pathways and metabolism to the N availability and providing new potential bioengineering targets for increased lipid accumulation.

## Results

### Physiological responses to nitrogen starvation and recovery in *Chlamydomonas reinhardtii* cells

*Chlamydomonas reinhardtii* cells show a high ability to adapt dynamically to environmental conditions. The stress adaption process is based in short- and long-term changes in metabolism affecting the morphological phenotype. Therefore, we have selected controls (0 h), three sampling times under N starvation (5 h-N5h, 24 h-N24h, 72 h-N72h), and two further samplings after N replenishment (77 h + N5h, 96 h + N24h), aiming to cover both short- and long-term responses for acclimation and recovery.

**Figure 1:**
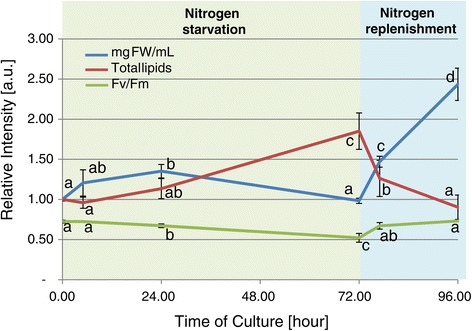
**Physiological measurements.** Variations in the photosynthetic rate (Fv/Fm) and in the accumulation of lipids, fatty acids, and fresh weight during the experimental time course. Different letters within series indicate significant differences (ANOVA followed by a TukeyHSD, *P* < 0.05). Fresh weight and fatty acid values were normalized as a relative abundance considering the average contents in T0. Fv/Fm values were plotted without transformation.

**Figure 2:**
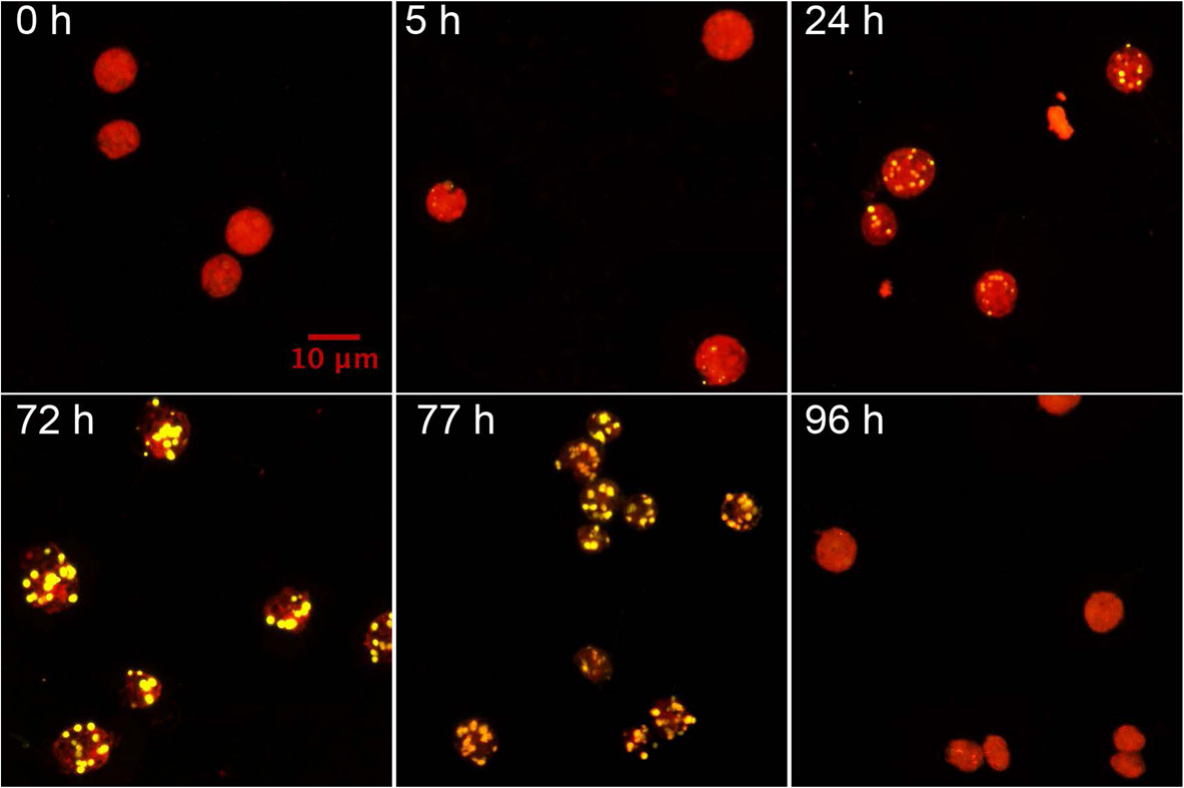
**Lipid bodies accumulation during the time course experiment.** Nitrogen starvation causes a quick accumulation of lipid bodies (yellow dots) with a maximum density obtained after 72 h of culture. Notice that the autofluorescence of the cells is dramatically reduced at 72 to 77 h, indicating a sharp decrease in the pigment content. Lipid bodies disappear completely 24 h after the addition of ammonia (96 h). Cells were fixed in 1% formaldehyde and stored at 4°C until analysis. Cells were stained with Nile red to identify the lipid bodies.

N starvation leads to a block of growth (Figure 1), which is significantly slower than that in control cultures (Additional file 1: Figure S1). The fresh weight (FW) of the cultures was sustained during initial N starvation, and reduced after 72 h. Also, chlorophylls were affected, with a turn in the culture color from green to yellow under N starvation with a 30% decrease in Fv/Fm (Figures 1 and 2). As expected, N starvation induced a quick accumulation of lipids, a 1.75-fold increase in 72 h (Figure 1), most of them in the form of lipid bodies (Figure 2). N starvation is affecting the normal physiological behavior of the cells, blocking cell growth and reducing photosynthesis. Cells stop dividing probably because there is no available N for sustaining protein and nucleotide biosynthesis. Under these circumstances intracellular N should be recycled to support critical life-supporting pathways. Photosynthesis and antennas are reduced to avoid oxidative cellular damages and changes in cellular pools. The excess of available energy and carbon is then channeled into an increased production of lipids. Lipids act not only as an energy and carbon sink, but also in membrane stabilization.

N replenishment quickly reverted these physiological adaptations, with total lipids, Fv/Fm, and chlorophylls returning to normal levels in 24 h (Figures 1 and 2). The FW of cell cultures was increased 1.5- and 2.5-fold at 5 h or 24 h after N replenishment. The degradation of TAGs and also the recovered photosynthetetic activity probably provided the energy needed for this fast recovery of vegetative cell growth. These results support the hypothesis that there are sensing mechanisms that regulate not only N uptake and N assimilation, but also many other metabolic pathways (ranging from glycolysis to secondary metabolism) defining physiology and cell growth.

**Figure 3:**
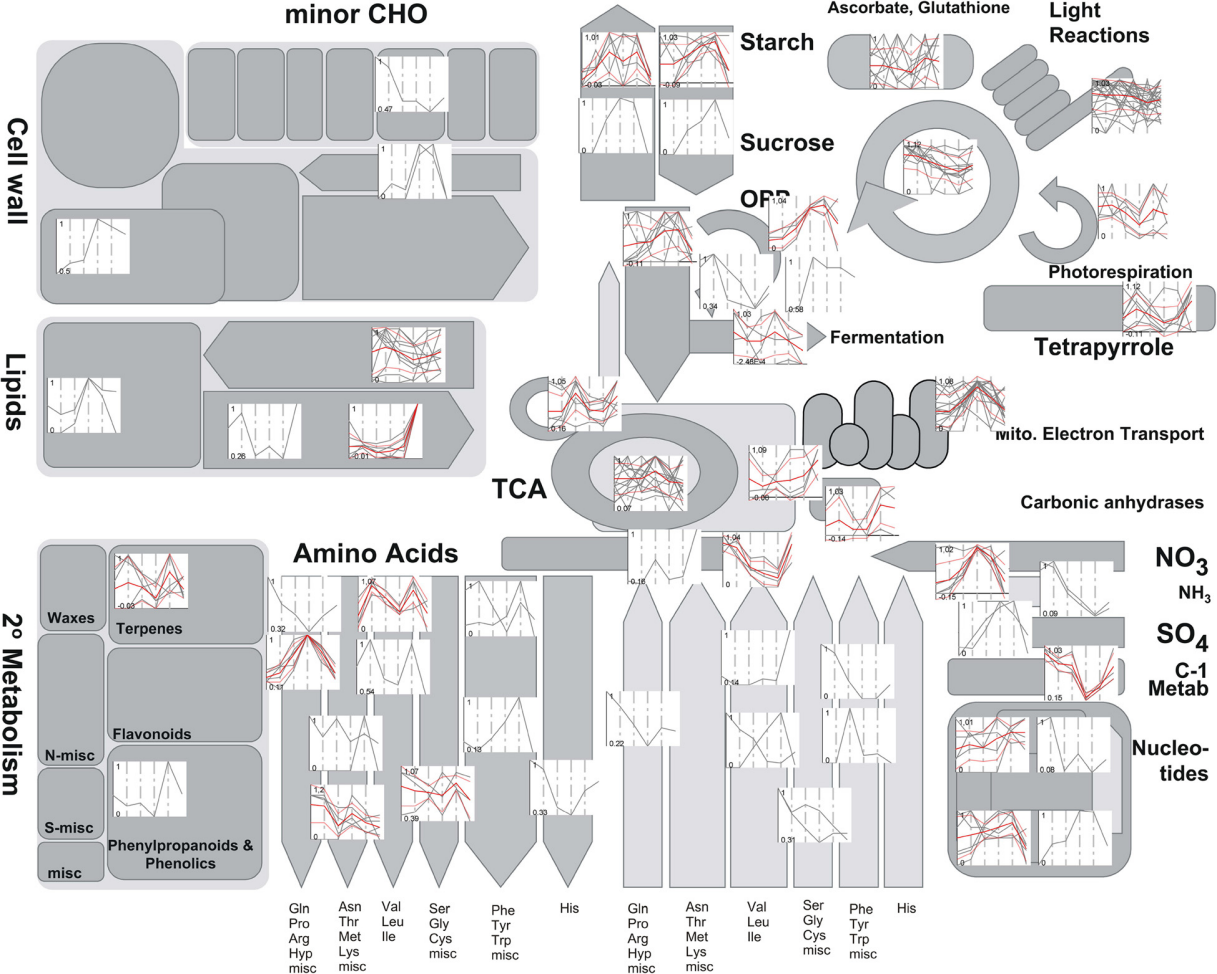
**Representation of N starvation- and recovery-induced changes in major pathways and processes using MapMan visualization platform and our** ***Chlamydomonas reinhardtii*** **mapping.** Lines in gray represent individual proteins that were differentially accumulated during the experiment (*P* < 0.05) at each category, thick red lines represent the average value within all of the clustered proteins at each time point, and thin red lines represent the average ± one standard deviation. Protein abundances were normalized as a percentage of the maximal value in the time series.

An initial functional overview of the changes in the proteome (Additional file 2: Tables S1, S2) and metabolome (Additional file 2: Table S3) showed that N starvation affects most of the cellular metabolism (Figure 3, Additional file 3: Figure S2, Additional file 4: Figure S3, Additional file 5: Figure S4, Additional file 6: Figure S5, Additional file 7: Figure S6), that is, ammonia transport and fixation proteins, photorespiration, or oxidative pentose phosphate pathways. Differential pathways are depicted below.

**Figure 4:**
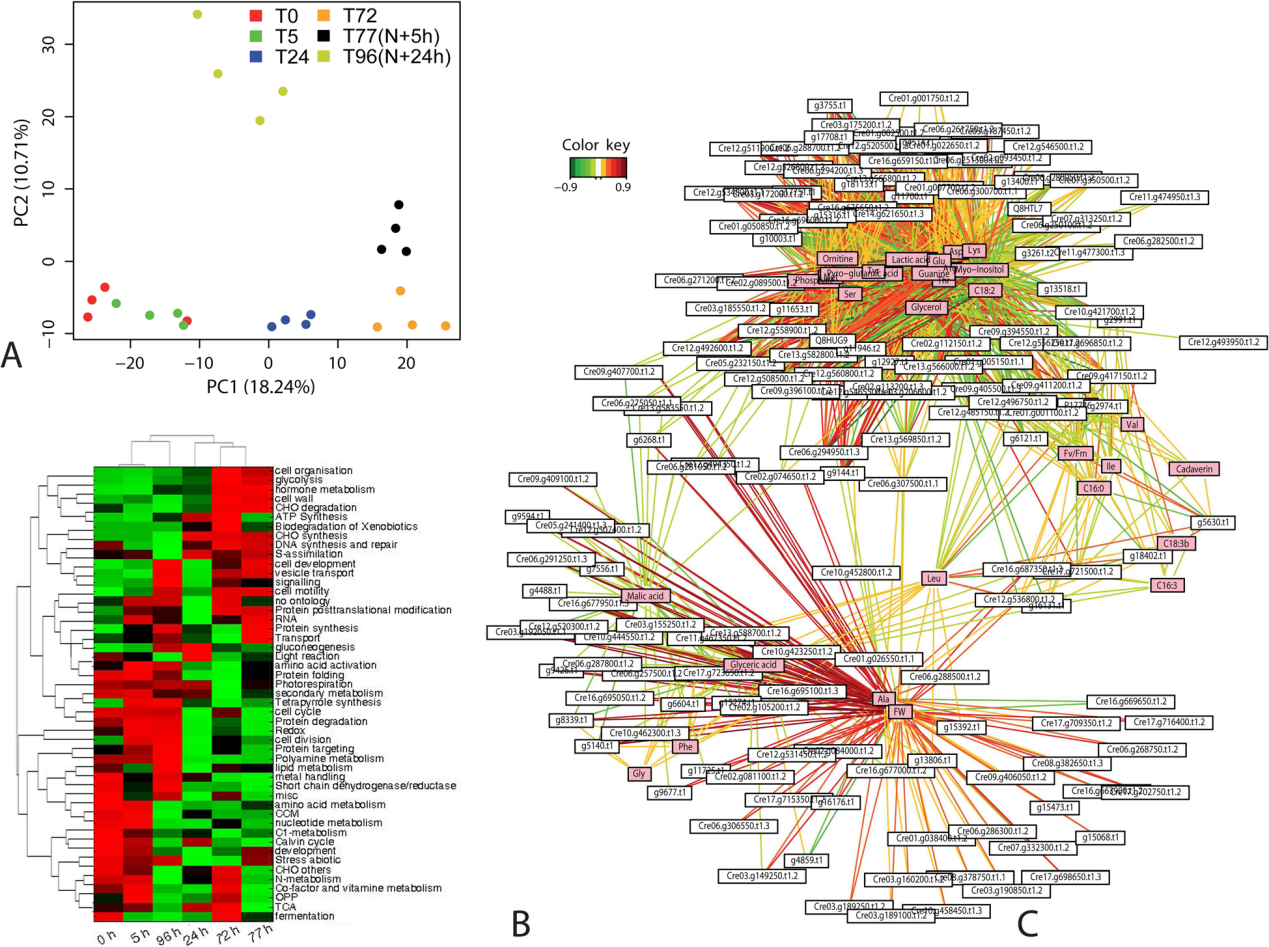
**Classification of the different samples according to multivariate methods.** **(A)** Principal componet analysis (PCA) of the integrated proteomic, metabolomic, and physiological datasets. Glutamate family enzymes (ASS, ASL, OCT, NAG), ammonia metabolism (Cre13.g592200.t1.2), purine biosynthesis proteins (Cre07.g318750.t1.2, Cre08.g364800.t1.2), NADH:ubiquinone oxidoreductase, cGMP-dependent kinases (Cre03.g199050.t1.2), glycolysis enzymes (PK, GAP-DH, PEPC), glyceraldehyde-3P-DH, glycerol, and C18:3 showed high correlations to PC1. Calvin cycle proteins (SBPase, PPE), chloroplastic ATPase, amino acid degradation, polyamine synthase, fatty acid elongation, catalases, and aspartic acid showed a negative correlation to PC1. Fresh weight, alanine, beta oxidation-related proteins (Acyl-CoA oxidases, HADH), oxidoreductases (Cre16.g677950.t1.3, g13806.t1, g4488.t1, g9426.t1), signal peptide and protein peptidases, and tetrapyrrole biosynthesis proteins showed a high correlation to PC2, while organic acids (fumaric and glyceric), phosphate, and photosynthesis-related enzymes (light reaction and carbon fixation), showed a negative correlation. These variables were used to infer the biological meaning of the principal components 1 and 2. Loading matrix is available in Additional file 2: Table S4. **(B)** Hierarchical clustering and heatmap of the analyzed proteins grouped by functional category according to MapMan. Three different clusters (0, 5 h, and 96 h, 24 h, and 72 and 77 h) can be distinguished, showing the different degrees of response to N starvation. The aggrupation of the 96-h and 5-h samples remarks the effect of the N repletion over the cultures. **(C)** sPLS-based network, showing the significant interaction between proteins and metabolites. Three major clusters can be distinguished, corresponding to fresh weight, glycerol/C18:2/C16, and N metabolism, which are mixed in the image. Fv/Fm and C16 are outside these groups acting as a link. For further details see Results and Discussion. This complex network is depicted in Additional file 6: Figure S5D, Additional file 10: Figures S9 and Additional file 11: Figure S10.

We applied multivariate analyses for integrating physiological measurements with protein and metabolite datasets, reducing the complexity of the data, and performing untargeted correlation network analysis [22,36-38]. Principal component analysis (PCA) and partial least square discriminant analysis (PLS-DA) resulted in a similar classification of the samples (Figure 4A; Additional file 8: Figure S7a, b; Additional file 2: Tables S4,S5) while sparse partial least square (sPLS) analysis gave a slightly different picture (Additional file 8: Figure S7c; Additional file 2: Table S6). PC1 seems to explain the adaption to N starvation: ammonia transport and assimilation enzymes are positively correlated to this component, while N demanding activities, like polyamine and protein biosynthesis, and amino acid degradation are negatively correlated. Furthermore, the high correlation of central metabolism enzymes and cGMP-dependent kinases showed that N starvation also affects normal respiration. The high correlation of glycerol pathways and C18:2 indicated the importance of lipid biosynthesis during N starvation. PC2, on the other hand, explains the N-starvation recovery and growth on the basis of an increased energy production through lipid degradation, and the recovery of the pigments biosynthesis. These findings support our previous hypothesis, based on physiological parameters, of how N starvation affects the cell function since the previously described effects can be explained at a biochemical level based on PC1 and PC2 (plotting other components does not improve the separation between groups (Additional file 8: Figure S7d, e, f)). Biclustering of functional categories of protein changes (Figure 4B) against the time points of N starvation and N readdition demonstrated impressively that the recovered cells are more similar to the control cells and that there is a complete remodeling of cellular processes during starvation and recovery. Clusters of short- and long-term responsive functional processes can be identified. Furthermore, we have used sPLS networks in which protein and metabolites were used as predictive variables to explain the physiological parameters aiming to establish interactions between proteins, metabolites, and phenotype (Figure 4C, Additional file 6: Figure S5D, Additional file 9: Figure S8, Additional file 10: Figure S9).

### Dynamics of 1,658 proteins and 52 metabolites during nitrogen starvation and recovery

The application of mass-spectrometry-based profiling of proteins and metabolites allowed a comprehensive analysis of the responses to N starvation and N readdition. More than 15,000 peptides and 3,200 proteins were detected in 552,529 spectra obtained from whole cell and nuclei protein extracts. 1,658 proteins were above the minimum abundance threshold for confident quantitation (Additional file 2: Tables S1 and S2). This number represents about 15% of the genes in the *C. reinhardtii* genome [16]. GC-MS allowed the unequivocal identification of 52 primary metabolites after comparison to reference standards (Additional file 2: Table S3) and quantified 60% of them with differential accumulation, at least in one of the sampling times.

The use of proteomics- and metabolomics-based approaches provides a direct readout of the metabolic and physiological adaptive mechanisms that are present in the cell and links molecular dynamics to genome-based theoretical metabolic networks [39,40]. The reconstruction of the *C. reinhardtii* network based on KEGG Orthology (KO) annotation consists in 7,330 reactions belonging to 263 pathways, which are catalyzed by 713 enzyme classes [23]. Of the total proteins described in this study, 845 were annotated to enzyme commission (EC) numbers using Biomart, the KEGG enzyme database, and manual annotation during the curation process. These enzymes defined 447 classes of reactions belonging to 157 pathways, constituting more than 50% of the modeled class reactions and pathways in *C. reinhardtii* [23,41].

We have further analyzed the total proteome using MapMan [30,42] and a custom bin map for the release 5.3 of the *Chlamydomonas* genome. A total of 1,233 proteins were assigned to functional bins. Employing this classification tool allowed us to plot the dynamics of the differentially accumulated proteins (*P* <0.05) to illustrate the overall changes in the metabolome (Figure 3), and also to focus specifically on photosynthetic (Additional file 3: Figure S2), N (Additional file 4: Figure S3, Additional file 5: Figure S4), lipid (Additional file 6: Figure S5), and nucleotide metabolism (Additional file 7: Figure S6). The integration of the generated data with the available datasets with transcriptomic [33] and metabolomic [34] data in the overlapping experimental points (0 and 72 h) allowed a robust and comprehensive analysis of the molecular changes induced by N starvation. However, 166 differentially accumulated proteins of the total protein dataset were not mapped to any pathway, indicating the need for a continuous improvement of functional annotation.

### Response of *Chlamydomonas reinhardtii* primary metabolism to N depletion and recovery

N starvation induces quick and profound changes in primary metabolism (Figure 3, Additional file 3: Figure S2, Additional file 4: Figure S3, Additional file 5: Figure S4, Additional file 6: Figure S5, Additional file 7: Figure S6). Glyceraldehyde-3-P-dehydrogenase (GAPDH) showed maximum peaks at 72 h. Interestingly, one pyruvate kinase isoform (Cre06.g280950.t1.2) was only detectable after N depletion, showing a drop in abundance 24 h after N repletion. At the same time pyruvate increased after N repletion. Fructose bisphosphate aldolase (FBA2) and glucose-6-P-isomerase (PGIC) showed a peak after 5 h and were then repressed until N repletion. This repression might lead to the accumulation of the glucose-6-P-pool which could be channeled into starch synthesis. Indeed, starch accumulates during N starvation [4,13]. Starch synthases (SS1, SS2, SS4, GBSS1) were upregulated during starvation, and quickly decreased after N repletion. The sugars fructose, glucose, and trehalose are accumulated during starvation (+15-, +5-, and +9-fold, respectively), being reduced after N repletion. Trehalose has been described as a substitute of N-containing compatible solutes [43] and a positive regulator of starch biosynthesis [44]. More recently a key role of trehalose in stress responses by regulating the SNF/bZIP system was postulated [45]. Glucose and fructose can also act as regulators of this system involving hexokinase (HXK) and potentially sucrose and trehalose metabolism [46]. However, sucrose metabolism is not clarified completely in *Chlamydomonas reinhardtii*. One gene which is at least assigned to sucrose metabolism is sucrose phosphate phosphatase (SPP). HXK and SPP abundances were already increased 24 h after N starvation, showing a maximum peak after 72 h, being quickly downregulated after N repletion.

On the other hand, the pool of glucose-6-P can also enter the oxidative pentose phosphate pathway. The rate-controlling enzyme of this pathway glucose-6-P-dehydrogenase (G6PDH) and 6-phosphogluconate dehydrogenase (6PGDH) showed an increased abundance under N starvation with a peak 5 h after N repletion (+3 to 5fold). In contrast, nonoxidative enzymes, transaldolase and cytosolic ribose-5-P-isomerase, showed an opposite trend with a minimum presence after 24 h of stress, potentially reducing the synthesis of ribulose-5-P (see discussion below).

**Figure 5:**
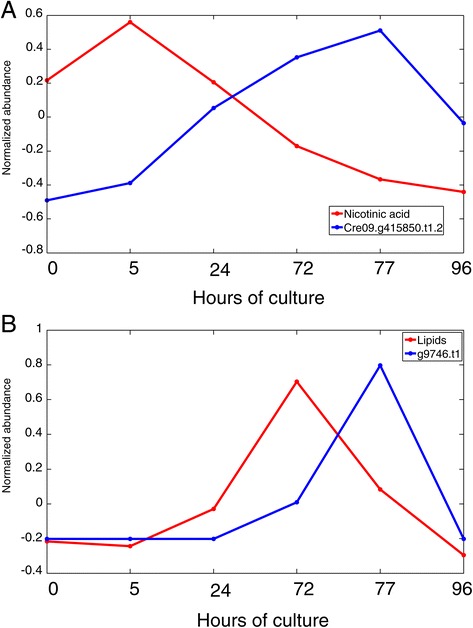
**Examples for identified Granger causalities.** Metabolite, protein, and physiological data were combined and analyzed with respect to time-shifted correlations using the toolbox COVAIN [38]. This procedure, called Granger causality, allows for the identification of directed correlations or nonlinear processes. Therefore, these associations must be carefully interpreted. **(A)** The metabolite nicotinic acid (NA) shows a time-shift behavior with respect to an NAD-reductase. Because NA is an intermediate in NAD biosynthesis, the increased levels of NAD-reductase may be involved in the consumption of NA. **(B)** A clathrin assembly protein involved in vesicle formation (COP II, see also Discussion) follows with a time-shift the accumulation of total lipids. After readdition of N, the protein concentration declines. This protein is also highly correlated to a major lipid droplet protein, glycerol, and various fatty acids, indicating its potential role in the formation of lipid bodies during N starvation (for further details see Results and Discussion sections).

To extend the classical correlation analysis, we applied Granger causality analysis (see also [22,38,47,48]). Here, time-lagged correlations are exploited with the potential that pairs of precusors and products can be identified. The Granger causality function is implemented in the COVAIN toolbox [22,49]. The COVAIN function also applies a Benjamini-Hochberg correction to reveal only significant correlations (for further information see the COVAIN manual; http://www.univie.ac.at/mosys/software.html). Because of the low number of time points, only a few Granger causalities have been identified and they have to be treated carefully. For example, in Figure 5A Granger causality showed a steady increase of NADH:ubiquinone reductase (Cre09.g415850.t1.2) that was negatively correlated with one of the precursors of NAD-biosynthesis nicotinic acid (Figure 5A), pointing to an increase in mitochondrial activity. The citric acid cycle (tricarboxylic acid, TCA) enzymes were in general upregulated, with a twofold increase for most of the proteins after 72 h, and a quick return to control levels after N repletion. Both succinate dehydrogenase (SDH2) and pyruvate dehydrogenase (PDH1) behaved the same. The abundance of TCA intermediates was not consistent with a unified response of this pathway. Pyruvate concentration is reduced during N starvation while malate is accumulated. Succinate and oxalate levels are constant during the whole experiment. The malic enzyme (ME3) also remains at constant levels during N starvation, but after repletion its abundance is increased sevenfold, which is correlated to a fivefold decrease of malate abundance. The upregulation of the TCA cycle is also functional for the production of carbon backbones for amino acid biosynthesis by anaplerotic reactions.

Most of the proteins related to oxidative phosphorylation are accumulated during N starvation. NADH-DH isoforms, ATP synthases, and cytochrome c oxidase (COX) increased up to seven-, two-, or eightfold, respectively (Additional file 2: Table S1). This response is not shared with other nutrient depletions such as sulfur [50] or temperature stress [51].

### N starvation induces a decrease in abundance of proteins involved in photosynthesis and increased carbon concentrating mechanism (CCM)

A sharp decrease of light-dependent reactions proteins (Additional file 3: Figure S2) followed N starvation. The reduction of cytochrome b6f complex (negative fivefold), ATP synthases (negative twofold), and photosystem II (PSII) proteins may explain at least partially the observed reduction in photosynthesis, confirming the observations of Majeran *et al.* [52], which showed a degradation of cytochrome b6f and light harvesting complexes (LHCs) after N starvation. Interestingly, although only two PSII proteins reached control levels, the Fv/Fm rate was fully recovered 24 h after replenishment. These results showed that, as proposed by Plumley and Schmidt [53], the efficiency of PSII during N starvation is not mainly limited by protein content but by other compounds such as chlorophylls. This was supported phenotypically and molecularly with a sharp recovery of green cells in the culture and the upregulation of the tetrapyrrole-related biosynthetic pathways (Figure 3).

Other pathways such as the cyclic electron flow have been recently described as essential mechanisms for photosynthesis [54] to provide ATP in situations of stress [55]. Cyclic electron flow-related enzymes showed a peak after 72 h (up to threefold), being then reduced after repletion, confirming its essential role as initially suggested by Peltier and Schmidt [56].

Most of the enzymes belonging to the CO_2_ fixation pathway, like RuBisCO large subunit (RBCL), FBPase, transketolase, or phosphoglycerate kinase, were reduced under N starvation (Additional file 3: Figure S2) as previously pointed out in [34]. Other proteins such as RuBisCO activase (RBCA) or phosphoribulokinase (PRKA) were increased with time, with a maximum abundance peak measured after 72 h in the case of PRKA.

Carbonic anhydrases (CAH1, CAH3, CAH7, and CAH8) belonging to CCMs are differentially regulated during N starvation. CAH3 decreased in abundance; conversely, CAH7 and CAH8 increased in abundance, peaking at 72 or 77 h. Phosphoenolpyruvate carboxylases (PEPC1, PEPC2) were not detectable when N was present in the media (0 and 96 h) and its maximum abundance was detected at around 72 h. Apparently, *C. reinhardtii* is highly flexible to maintain CO_2_ fixation, triggering specific enzymes depending on the type of stress, as cold stress was characterized by increased PEPC2 and CAH3 [22].

We have not detected a good correlation between changes of photosynthesis-related proteins and its transcripts (r^2^ = 0.166), showing that the specific regulation of protein species abundance does occur at the translational level, but also by posttranslational and degradation mechanisms.

### Nitrogen metabolism quickly adapts to an environment without ammonia

N starvation leads to an increased abundance of specific mRNAs, proteins, and metabolites [15,33,34,57]. In our study we used the strain CC503, a *nit1 nit2* mutant lacking an active nitrate signaling pathway (*nit2*). As expected, no differences within nitrate transporters, nitrate, and nitrite reductases (Additional file 4: Figure S3) were detected. By contrast, proteins involved in NH_4_^+^ assimilation were overaccumulated: two ammonia transporters (g3261.t2, Cre13.g569850.t1.2) were induced by N starvation. Interestingly, Cre13.g569850.t1.2 accumulation continued with a maximum abundance 24 h after N repletion (a threefold increase compared to 24 h of N starvation), indicating a long responsive time in this element. Enzymes of the Glu-Gln (GS/GOGAT) system were also upregulated, and the abundance of all glutamine synthase (GS) isoforms increased five- to tenfold, just to decrease quickly to control levels after N repletion. On the other hand, glutamate synthase (GSN) isoforms behave differentially: GSN1 abundance is stable, while GSN2 was induced by N starvation. Glutamate dehydrogenases (GDH1, GDH2), involved in N degradation, showed a tenfold decrease starting to respond to N repletion after 24 h (slower than GS and GSN enzymes). The correlation of fold change at transcript and protein levels (r^2^ > 0.8) points to the transcriptional regulation of this system.

The central amino acid metabolism and some degradation pathways were decreased (two- to threefold) after N starvation (Figure 3). Conversely, the glutamate family pathway quickly responded to the availability of N, being strongly upregulated by N starvation with a fourfold increase in N-acetyl-gamma-glutamyl-phosphate reductase (AGPR), arginosuccinate lyase (AGS1) and synthase (ARG7). Furthermore, three enzymes of the branched amino acid biosynthesis (acetolactate synthase 1 and 2, ALS1, ALS2; acetohydroxy acid isomeroreductase, AAI1) showed abundance peaks at 5 h and 77 h. Most of the amino acids were tightly connected in the sPLS correlation network (Additional file 11: Figure S10) with glutamate and its derivative pyroglutamic acid among the central nodes. These nodes also linked proteins involved in a wide range of activities and functions, showing the diversity of the responses triggered by N starvation.

### Cytosolic ribosomes are accumulated during N starvation, while chloroplastidic ribosomes are degraded

A general ribosomal degradation and resynthesis is classically associated to N starvation [58]. These observations are partially supported, at least for chloroplast ribosomes, by a recent high-throughput transcriptomic study [33]. We detected a differential behavior between chloroplast and cytoplasmic ribosomes (Additional file 5: Figure S4). Evidence of protein accumulation was observed for cytosolic ribosomes, with a two- to tenfold increase in the proteins corresponding to 40S. In the case of chloroplastidic ribosomes, the abundance of both subunits decreased after N starvation, being downregulated by more than 40% of the quantified proteins. However, these proteins quickly responded to N replenishment, recovering its initial abundance values in 5 h. Our data refine the current model of ribosome recycling following N starvation, in contrast to previous theories pointing to an untargeted ribosomal degradation-resynthesis. Here, we rather observe a targeted accumulation of cytosolic and a degradation of chloroplastidic ribosomes. Furthermore, the differential behavior of cytosolic, increased, and chloroplast, decreased, ribosomes suggests a more active role of the nuclear encoded proteins for adapting to N starvation.

### Response of lipid metabolism to N starvation

N starvation led to the accumulation of lipids (Figures 1 and 2) mainly in the form of TAGs [10]. In consequence we expected to detect upregulated lipid biosynthetic pathways but, surprisingly, most of the enzymes of the fatty acid biosynthesis were not changed after starvation (Additional file 6: Figure S5A). The key enzymes 3-ketoacyl-CoA-synthase 1 (KAS1), which catalyzes the reaction of acetyl-ACP with chain-extending malonyl-ACP, and one isoform of enoyl-ACP-reductase (Cre06.g294950.t1.3), which catalyzes the last step of the fatty acid biosynthesis, are downregulated. This behavior was previously shown at protein level [59]. The downregulation of two triglyceride lipases (Cre01.g002400.t1.3, g9707.t1) suggests that this decrease compensates the decrease in lipogenic enzymes allowing an effective lipid accumulation. At transcript level a modest increment (less than +2-fold) of biosynthetic genes of fatty acids under N deprivation was observed [33]. An undescribed monogalactosyl diacylglycerol synthase annotated to locus g14367.t1 showed a +3-fold change following N starvation. The product of this reaction could serve as a substrate for the desaturation of oleic and linoleic acid [60] while also controlling the cell proliferation by blocking replicative DNA polymerase as described in animals [61]. The pool of glycerol, essential for the formation of TAG, was significantly increased after N starvation (+4-fold; Additional file 2: Table S3), and can be explained by the accumulation of glycerol-3-phosphate dehydrogenase/dihydroxyacetone-3-phosphate reductases (GPD2, GPD4) (Additional file 6: Figure S5B). These enzymes showed a high correlation to glycerol in the interaction network (Additional file 6: Figure S5C). This network also links acetate, glycerol, and C18:2, oxidoreductases, ribosomal proteins, and a major lipid droplet protein (Cre09.g405500.t1.3) [62,63]. This protein has a high similarity to a major protein in lipid droplets in *Haemotococcus pluvialis* and *Dunaliella* (BLAST e-values of 10^−84^ and 10^−46^, respectively, NCBI-nr database). Cre09.g405500.t1.3 was not detected at 0 h, but from 5 h to 72 h it increased its abundance up to 14-fold. Proteins for NH_4_^+^ transport and assimilation and specific signaling proteins (BSU1) were also part of this network. These results demonstrate the power of the integrative networks based on sPLS-correlation for discovering new interactions between proteins and metabolites, allowing the capability to also accurately associate uncharacterized proteins to functional clusters.

N starvation leads not only to the accumulation of lipids, but also to the change of the total lipid composition of the cells. We have studied 10 long chain fatty acids (Additional file 2: Table S3, Additional file 6: Figure S5D). Four of them, C16:3, C16:0, C18:2, and C18:3 (9,12,15), were significantly different in our experiment. C16:0, C16:3, and C18:2 were accumulated, while C18:3 (9,12,15) was reduced. Chloroplastidic desaturase ∆12 (CDD12, Cre13.g590500.t1.2) is eightfold reduced at transcript level, and may explain the reduction in C18:3. Stearoyl-CoA desaturase ∆9 (SCD, Cre09.g397250.t1.2) transcripts are twofold increased during N starvation and may also explain the increase of C18:2. How this system is regulated is not clear, and cannot be elucidated alone at the protein or transcript level.

N replenishment causes a quick activation of beta-oxidation pathways, with increased levels of five acyl-CoA-oxidase isoforms and also the recovery of the levels of the lipase Cre01.g002400.t1.3.

### Nitrogen starvation induces significant changes in the nuclear proteome

To further investigate changes at the nuclear regulatory level we first studied nuclei-enriched fractions of *C. reinhardtii* (Additional file 2: Table S2). Secondly, we performed an *in silico* analysis of total cell and nuclear fractions focusing on nuclear proteins related to signaling and transcriptional regulation and finding 268 proteins (Additional file 2: Table S8), from which 136 were differentially expressed (*P* ≤0.01). As expected, the levels of histones and other core proteins did not significantly change with time, whereas other chromatin-interacting proteins followed different dynamics. N starvation induced an initial decrease in RNA polymerase I and RNA helicases, which were partially recovered after repletion. On the other hand, N starvation triggered the accumulation of DNA binding proteins, such as Cre06.g252000.t1.2, a leucine/zipper transcription factor, which was quickly silenced again after N replenishment (a fivefold reduction in 5 h). An Argonaute-like protein (AGO; Cre04.g214250.t1.3) was also induced by N repletion, suggesting a role of the siRNA system [64]. The DEAD/DEAH box helicase Cre01.g021600.t1.2 is also accumulated in the absence of N in the medium, indicating that some SWI/SNF regulation pathway may be occurring. This class of enzymes and regulation has been described to be part of the abiotic stress response mechanism [65]. We have used the Plant Transcription Factor database [66] to mine the *Chlamydomonas* genome release 5.3 for finding transcription factors. However, many of these postulated transcription factors were not detected in the nuclear-enriched fractions, pointing to its low abundance and the technical challenges for high-throughput identification. To date most of these proteins are uncharacterized, and even for the known families their members can have different regulatory roles [67].

N starvation is known for triggering gametogenesis and sexual reproduction in *C. reinhardtii* [31], but we have not detected related proteins, probably because they are expressed at very low levels or they are not functionally annotated.

## Discussion

*C. reinhardtii* cells respond to changes in the availability of ammonia by drastically changing their metabolism and normal development. In this work we analyzed the changes in the metabolome and proteome, also integrating other available datasets, thus presenting the most complete and comprehensive overview of the response to total available nitrogen and its recovery. The design of this study, together with the employed analytical techniques, allowed us to update current knowledge, providing new insights for the understanding of the complex metabolic dynamics that follow N starvation and, for the first time, recovery of vegetative growth after N replenishment, by using high-throughput omics analyses.

### Nitrogen/carbon balance defines the metabolic switch under N starvation

The sensing of the balance between N and C is transduced into a change in N- and C-responsive pathways, leading to a metabolic adjustment that dominates many pathways and defines the observed physiological and phenotypical changes. However, it has to be considered that the presence of acetate in the medium will change the cellular sensitivity towards the maintenance of favorable C/N ratios [68], causing increased responses to the oxidative stress as a side effect of the downregulation of photosynthesis and carbon assimilation [69]. N starvation also leads to increased ammonia uptake and assimilation proteins and a decreased nucleic acid and protein biosynthesis, which may be the cause for the reduced cell growth.

The reduced photosynthesis is proposed to be mediated by the degradation of LHCs and cytochromes, rather as an adaptive mechanism to stress and energy contents than as a way to recycle nitrogen. The reduction of PSII activity would lead to the production of less reactive oxygen species, but at a cost of reducing ATP and NADPH production. But even in a situation of reduced growth, as observed under N starvation, energy is still necessary to sustain metabolism. We detected an increase of cyclic electron flow proteins which pump H^+^ to the thylakoid lumen in order to increase ATP production. Furthermore, and based on the metabolic reconstruction, an increased pool of glucose-6-P was predicted. Part of this glucose-6-P can join the initial reactions of the oxidative pentose pathway. The corresponding enzymes were accumulated, rather than glycolytic enzymes. In contrast, nonoxidative enzymes, transaldolase, and cytosolic ribose-5-P-isomerase showed an opposite trend with a minimum presence after 24 h of stress, potentially reducing the synthesis of ribulose-5-P. The pool of ribose-5-P could enter the chloroplastic reductive pentose phosphate pathway to produce 3-phosphoglycerate, which can continue with the glycolytic pathway. The result of this reprogramming could be the production of enough pyruvate and NADPH + H^+^ to support biosynthetic processes at a cost of half ATP payback compared to glycolysis. This hypothesis was supported by the accumulation of phosphoribulokinase (PRK1), which followed the same abundance trend as G6PDH and 6PGDH. These changes in protein abundance are coincident with the changes in the mRNA expression levels given by [33] in an N-depletion study; furthermore, [34] recently described the increased accumulation in the metabolites 3-phosphoglycerate and ribose-5-P during N starvation. This remodeling, although energetically unfavorable, can be used by the cell to maintain adequate levels of NADPH + H^+^, which are required to maintain an increased lipid production. However, future studies applying flux analyses are necessary to investigate these processes in more detail.

The functional role of cyclic electron flow and the quick recovery of PSII efficiency (correlated rather to pigment biosynthesis than to the recovery of PSII proteins) indicate that photosystems and electron transfer chain were fully functional during N starvation, even though the abundance of some subunits and cytochromes was reduced.

Athough CO_2_ fixation enzymes were in general reduced, the abundance of CCM proteins CAH7, CAH8, and PEPC1 increased. CAH7 was only detected after a long exposure to N depletion (72 h) and may explain why this enzyme was not detected in previous studies limited in time to not more than 48 h [33,34]. CAH7 and CAH8 are similar and both of them contain a hydrophobic chain which localizes them in periplasmic space associated to the plasma membrane, probably covering decreased CAH1 and 2 activities and transferring inorganic CO_2_ to a transport system or pore [70]. The specific role of these CAHs under nutrient deprivation remains undetermined and cannot be easily explained at this time. By contrast, the kinases belonging to the Snrk2-SNF2 family have an essential role in the control of gene expression through the activation of bZIP transcription factors and SWI/SNF chromatin remodeling complexes [71,72]. These were quickly responsive to N starvation in our experimental system. Kinases of this family control the lipid accumulation in yeast [73], and have been linked to increased tolerance to N deficiency in *Arabidopsis* [74] or S in *C. reinhardtii* [50]. SNF families, also implied in energy sensing and gene regulation [75], control the metabolic response to other stresses like cold acclimation, inducing the accumulation of sugars and starch [51]. The accumulation of the signaling molecules glucose-6-P and trehalose [45,76] and the specific dynamics of HEX and SPP found in the N starvation, provide an extra support to this hypothesis.

### Branched chain amino acids linked N metabolism and cell growth

The unavailability of N caused a rearrangement of amino acid metabolism at the same time. The pool of glutamate, essential for ammonia assimilation, was drastically reduced after starvation, triggering aspartate aminotransferases and citrate synthases from the oxoglutarate pathway. Enzymes belonging to branched chain amino acids (BCAs) were negatively correlated to the abundance of Val, Ile, and Leu, which indicates that enzyme abundance is increased to compensate the lower abundance of BCAs. Leu and Ile were significantly correlated (r^2^ > 0.9) to Cre16.g687350.t1.2, an acyl-CoA oxidase and mediator of amino acid degradation, and a monofunctional catalase which may be controlling the oxidative damage. Interestingly, the same interaction cluster was found when analyzing the sPLS-based network; it was the bridge that linked FW and amino acid-nitrogen clusters.

The increased abundance of carbamoyl phosphate synthetase subunits (CMP1, CMP2) and the dynamics shown by arginine and ornithine suggest the importance of the urea cycle for recycling the ammonia originated in the protein breakdown.

### Are glycerol metabolism, MLDP, and COP II proteins key elements for lipid droplet formation and TAG accumulation?

The increased abundance of total lipids and lipid droplets following N-deprivation could indicate that the TAG of the droplets is synthesized de novo. The expected increase of the fatty acid biosynthetic pathway was, in general, not present but two Acetyl-CoA-Synthetases (g1224.t1, Cre12.g507400.t1.2) required for the activation of fatty acids and forming part of lipid droplets [62] were significantly accumulated. Glycerol metabolism, needed to provide the backbone for the glycerolipid biosynthesis, was significantly responsive, with a combined accumulation of the proteins of this pathway of more than 50-fold. Cre09.g405500, a major lipid droplet protein (MLDP) [62,63], increased 14-fold under N starvation and was linked to C18:2 and glycerol, showing further correlation (>0.93) with clathrin and COP II, which forms vesicle coats and allows liposome fusion [77]. The MLDP protein was identified by Moellering and Benning and by James *et al.* after analyzing lipid bodies associated proteins [62,78]. Recently Wase *et al.* described a similar behavior of this protein under N deprivation, showing a 4.1-fold change after six days of culture without nitrogen [59]. MLDP is highly correlated with lipid accumulation during N starvation and dropped completely after N replenishment. MLDP and other proteins linked by sPLS and Granger causality analysis such as COP II provide hub proteins in the adaptation process to N starvation and revovery (see Figure 5B). These proteins are involved in vesicle formation, suggesting their strong role in lipid body formation, thereby confining the TAGs into storage structures and promoting their accumulation. Lipid body formation can be also influenced by GTPases as COP II protein is inactivated by GTPases [79], which we also found to be decreased during N starvation. Correlation networks need to be further analyzed since they also reach fatty acid and starch biosynthetic enzymes, pointing to key processes and promising targets for strain engineering.

The increased C18:2 and the consequent alteration of the different fatty acids suggest a remodeling of the membranes, and thus fatty acids in membranes seem to be recycled into TAGs. TAG lipases and phospholipases were downregulated.

N repletion led to an increased beta-oxidation for rapid lipid degradation and also increased the abundance of five acyl-CoA-oxidases. These were not correlated to enzymes of the BCA metabolism, demonstrating differential regulatory pathways. The lipase Cre01.g002400.t1.3 that was recovered after N replenishment was negatively correlated (>|0.9|) to the translation initiation inhibitor (Cre12.g551350.t1.2) and a protein showing a DNA methyltransferase domain (Cre12.g508050.t1.2). This might indicate that the transcriptional response to N starvation is regulated not only by transcription factors, but also by epigenetic mechanisms, as suggested by [50].

### Can N recovery help us to understand lipid accumulation or metabolism and provide new targets for biotechnological improvement of oil production?

The study of specific changes occurring 5 h and 24 h after N resupply provides specific targets that could have potential use for bioengineering applications. Some of them have been depicted above (PEPC, CAH); however, there are specific targets that can be highlighted. One of the possible ways of intervention involves the enzymes and proteins related to lipid body stabilization and degradation with MLDP a key protein in the lipid biogenesis network. MLDP has been studied extensively and, despite its silencing by RNAi, does not lead to an increased lipid accumulation; however, the droplet size is significantly increased [62]. This is a surprising result since our data, and also that of [54], points out that the abundance of this protein is positively correlated to lipid accumulation. The combined manipulation of this protein together with beta oxidation, silencing acyl-CoA oxidases (especially Cre16.g687350.t1.2, Cre11.g467350.t1.2) and lipase (Cre01.g002400.t1.3), could result in an increased accumulation of lipids. Lipid transporters also play a key role in lipid metabolism, with Cre15.g641200.t1.2 a mitochondrial fatty acid transporter as a responsive candidate. g13764.t1, Cre13.g573150.t1.3 (hydroxylases) and Cre11.g467350.t1.2 (acyl-CoA oxidase) were accumulated (hydroxylases) or repressed (acyl-CoA oxidase) under N starvation, following the same trend as its transcripts.

Interestingly, these genes were not reported to be significantly expressed under other stresses such as C, S, or Fe deprivation or oxidative stress according to AlgaePath [80]. However, finding central metabolism enzymes related to lipid biogenesis and only responsive to N starvation (when comparing with the available NGS/proteomics datasets) is particularly difficult. Furthermore, modifying the gene expression of a number of genes is tricky, and a perfect flux model should be available in order to avoid unintended effects. Previous attempts to increase lipid accumulation by targeting specific genes have not been completely successful [10]. This indicates that lipid accumulation is the result of a complex regulatory network linking cellular processes such as vesicle formation as well as processes of central metabolism such as G6P, branched chain amino acids, and energy metabolism. This is clearly demonstrated by the system-level analysis of our study.

In this sense, our results and other proteomic and transcriptomic datasets [23,33-35,50,59] show many complementary genes/protein clusters responsive to stress [80]. This suggests that there are major points of metabolic regulation based on common signaling elements that can be considered as potential targets for increasing lipid accumulation. Numerous DNA and RNA binding proteins and helicases showed differential accumulations in our assay (described above), but these candidates could not be easily proposed as biotechnological targets since their interaction network is unknown. On the other hand, the BRI1 suppressor (Cre01.g050850.t1.2), an inhibitor of BRI1, a receptor-like kinase which is responsive to brassinosteroids located both in plasma and in nuclear membranes [81], was correlated to glycerol and C18:2, and it is known to initiate a signaling cascade leading to regulation of gene expression in the nucleus through BZR/BES proteins [82]. BZR is known to block the metabolic switch in response to P deprivation [83] and other abiotic stresses [82] in *Arabidopsis* and also inhibits chloroplast development [84], so a similar effect could be expected in *Chlamydomonas*. Thus, blocking not only the receptor, but also the transcription factor BZR by 14-3-3 proteins may be needed to respond to N starvation. In our dataset a 14-3-3 protein responding to N deprivation and recovery was quantified (Cre06.g257500.t1.2). The BRI1 suppressor was only detected when N was not present in the medium, showing a quick adaptive response to environment.

FtsH chloroplast metalloproteases are closely related to development [85], stress responses [86], and chloroplast function [87], also regulating lipid degradation in bacteria [88]. We found three metalloproteases (Cre12.g485800.t1.2, Cre17.g720050.t1.2, g14586.t1) that were downregulated when N was absent from the media. Interestingly, and according to [74] and considering all available datasets, Cre17.g720050.t1.2 responds only to N starvation, making this enzyme a potential candidate for further study.

## Conclusions

The comprehensive analysis of systemic responses to N starvation and recovery in *C. reinhardtii* demonstrated that metabolism and growth are significantly affected at a system level. A complex network of stress-responsive proteins, metabolites, and physiological parameters was established, expanding our current understanding of physiological processes driven by a small set of proteins. Many uncharacterized proteins were identified by multivariate correlation network analysis to be involved in the response to N starvation. By N readdition it was possible to extract a list of proteins that showed a fast recovery effect, suggesting that they are highly involved in the re-establishment of vegetative cell growth. This study provides new insights and alterations to previous models and offers a complex dataset, which will be further analyzed towards increasing our biochemical understanding of the adaptive mechanisms to N starvation and recovery in *Chlamydomonas*.

## Methods

### Strains and cultures

*Chlamydomonas reinhardtii* CC-503 *cw92,* mt+, agg1+, *nit1*, *nit2* (available at the Chlamydomonas Culture Collection, Duke University) cultures were grown in HEPES-Acetate-Phosphate medium supplemented with 7 mM NH_4_Cl (HAP + N; TAP medium in which Tris was replaced by 5 mM HEPES) at 25°C with shaking (120 rpm) in a 14:8 light:dark photoperiod (85 μmol m^-2^ s^-1^; Sylvania GroLux lamps). Cultures were pelleted down by centrifugation and resuspended in HAP -N (NH_4_Cl was replaced by 7 mM KCl) media to a final density of 1-3 × 10^5^ cells mL^-1^. Cells were sampled at times 0, 5, 24, and 72 h. After this sampling, NH_4_Cl was added to the HAP-N cultures to a final concentration of 7 mM, and then the cultures were sampled after 5 h (77 h) and 24 h (96 h).

### Physiological measurements

At each harvesting time the cell density was measured by employing a Thoma counting chamber and the fresh weight was determined gravimetrically. The photosynthetic rate was measured with an imaging/pulse-amplitude modulation fluorimeter (OS1-FL, Opti-Sciences).

Total lipids were extracted from frozen pellets with 200 μL of a mixture of chloroform:isopropanol (1:1) and vigorous vortexing for 3 min. The samples were centrifuged (14.000 × g, 5 min, room temperature) and the supernatants were transferred to a new tube. The pellet was re-extracted with 500 μL of hexane and vigorous vortexing for 3 min. The samples were centrifuged, and the combined supernatants were dried in a speed vac. The amount of lipids was determined gravimetrically.

### Microscopy

The *Chlamydomonas* cultures were fixed in 3% (v/v) formaldehyde and kept at 4°C until staining. The cells were stained in a mixture of HEPES-Acetate-Phosphate medium with 3% (v/v) formaldehyde and 5 μg mL^-1^ of Nile red and incubated 15 min in the dark before imaging. Nile red was freshly added to the staining solution from a concentrated stock (0.1 g mL^-1^ in acetone). The stained cultures were directly observed by adding 15 μL to each slide under an LSM 780 confocal microscope (Zeiss, Germany) and Z series covering all of the cell height were captured. DIC, Nile red, and autofluorescence were recorded in different channels. From these stacks the four images closer to the middle section were selected and used for obtaining a maximum projection using the software Fiji (Figure 2).

### Nuclei isolation

Nuclei were isolated following the protocol described in [89] starting from 250 mg of fresh weight. Isolated nuclei were separated from cell debris by the use of a Percoll cushion.

### Quantitative proteome analysis (GeLC-LTQ-Orbitrap-MS)

Proteins were extracted from frozen pellets (40 to 50 mg fresh weight), gel fractionated, and trypsin digested following a previously described protocol [18]. Ten micrograms of digested peptides were loaded per injection into a one-dimensional nano-flow LC-Orbitrap/MS and resolved in a 90-min gradient from 5 to 40% (v/v) acetonitrile/0.1% (v/v) formic acid using a monolithic C18 column (Chromolith RP-18r, Merck, Darmstadt, Germany). MS analysis was performed on an Orbitrap LTQ XL mass spectrometer [22].

The raw data were searched with the SEQUEST algorithm present in Proteome Discoverer version 1.3 (Thermo, Germany) as described by Valledor *et al.* [17] using the *Chlamydomonas* genome v.5.3 (17,737 accessions), *Chlamydomonas* mitochondria (8 accessions), and chloroplast (76 accessions) databases. Only highly confident proteins, defined by at least two peptides with XCorr value greater than charge state +0.25 and 5% FDR, were considered for this work. Protein functions were identified using the BioMart tool available at Phytozome (http://www.phytozome.org), Mercator (http://MapMan.gabipd.org/web/guest/app/mercator), and the Algal Functional Annotation Tool [28].

The identified proteins were quantitated by a label-free approach based on total ion count followed by an NSAF normalization strategy:

$$ {(NSAF)}_k=\raisebox{1ex}{${\left(PSM/L\right)}_k$}\!\left/ \!\raisebox{-1ex}{${\displaystyle {\sum}_{i=1}^N}{\left(PSM/L\right)}_i$}\right. $$

in which the total number of spectra counts for the matching peptides from protein k (PSM) was divided by the protein’s length (L), then divided by the sum of PSM/L for all N proteins within each sample. Before quantitation a second filtering step was performed to retain only those proteins with enough abundance (minimum abundance of 0.001) for a low-biased quantitation [90,91]. Statistical analyses were conducted according to [92,93]. Proteins were accounted for quantification only if they were present in all of the biological replicates (n = 4) at least one sampling time, or in five samples corresponding to different times. Missing spot volumes were determined from the dataset employing a sequential K-nearest neighbor algorithm. Univariate comparison between treatments was performed by the nonparametric Kruskal-Wallis test over log-transformed data for obtaining *P*-values (Additional file 2: Tables S1, S2, S8). Protein abundance values were scaled and subjected to principal component analysis (PCA), discriminant analysis (PLS-DA), sparse partial least square (sPLS), and heatmap-clustering analyses (Additional file 2: Tables S4-S6).

### GC-MS polar metabolite and fatty acid methyl ester (FAME) analyses

Polar metabolites were extracted from frozen pellets (15 to 20 mg fresh weight) as described in [94]. Fatty acids were extracted from frozen cell pellets (15 to 20 mg) previously disrupted with liquid nitrogen and glass beads in a homogenizer (15 seconds, maximum speed, Retsch Mixer Mill MM400). One mL of cyclo-hexane:water (1:0.4) was quickly added to the homogenized tissue, and mixing was repeated with the same conditions. The organic phase was recovered after centrifugation (24,000 × g, 10 min) and transferred to a new tube. Pellets were re-extracted following the same procedures, and both organic fractions were combined in a 2-mL tube and dried by speed vac.

Sample derivatization (polar metabolites), methylesterification (lipids), and GC-MS measurements were carried out following the procedure previously developed in our group [95] on a triple quad (TSQ Quantum GC; Thermo) instrument. Metabolites were identified based on their mass spectral characteristics and GC retention times, by comparison with retention times of reference compounds in an in-house reference library. Only metabolite peaks that were detected in all of the biological replicates for at least one sampling point or in more than 18 samples were further considered. Normalized abundance values for each metabolite were obtained by dividing peak areas by the total peak areas of each sample (sample-centric normalization). Significant differences between sampling times were assessed by One-way ANOVA (Additional file 2: Table S3), and multivariate analyses were conducted together with proteins and physiological data as described above.

### Bioinformatic tools

All statistical procedures described above were performed using the software R v2.15.2 [96] core functions plus the packages SeqKNN, mixOmics, and gplots. Confocal microscopy images were processed and analyzed using the last version of the software Fiji [97] employing core packages. MapMan 3.5.1R2 [42] was used to make a functional classification of the proteins. A map for the proteome of the JGI_236 version was specifically created using Mercator [23] and the Algal Functional Annotation Tool [28]. The accuracy of the classification was supervised by comparison to local databases, also adding some manual annotations. Experimental data were classified according to this newly developed map.

We have used the dataset of transcriptomic data provided in [33]. This dataset was generated using version 4 of the *Chlamydomonas* genome. To compare this dataset to ours, we have BLASTed Chlamy 4 against Chlamy 5.3 considering significant all hits with e-values lower than 10^-80^. The equivalences between Chlamy 4 and Chlamy 5, as the Illumina-based normalized abundance employed for this work, are given as supplementary material (Additional file 2: Table S9).

Granger causality was tested with the statistical toolbox COVAIN [38]. Parameter settings for COVAIN were Granger lag time 1 and Granger significance *P*-value 0.05 including a Benjamini-Hochberg correction. A list of all identified Granger causalities can be found in Additional file 2: Table S10.

## Additional files

Additional file 1: Figure S1. Comparison of cell growth (fresh weight) of N repleted and depleted cultures of *C. reinhardtii* CC503 grown under the same conditions employed in this study. Cultures were grown in triplicate. Additional file 2: Table S1. List of the 1,534 quantified proteins in whole cell fractions. Protein abundance was determined following an NSAF approach. The mean abundance ± SD for each sampling time is indicated, as well as *P*- and q-values. Percentage of coverage and number of unique peptides used for identification and score are indicated. Deflines, E.C. number, and MapMan bins were manually curated. **Table S2.** List of proteins extracted from nuclei-enriched extracts. Spectra were processed as previously described. Deflines, E.C. number, and MapMan bins were manually curated. **Table S3.** List of the 52 quantified metabolites. Metabolite abundance was estimated from the peak areas of the indicated characteristic ions. The mean normalized abundances with respect to control ± SD for each sampling time are indicated as well as *P*-values. **Table S4.** PCA summary, sample scores, and variable loadings of the integrated (proteins, metabolites, physiological measurements) dataset. **Table S5.** PLS-discriminant analysis correlations of the integrated dataset. **Table S6.** sPLS analysis correlations of the integrated dataset employing proteins-metabolites as predictive variables and physiological measurements as responsive variables. **Table S7.** Values of individual replicates of the datasets employed for multivariate analyses. (a) Normalized abundances for proteins; (b, c) normalized abundances for polar metabolites and FAME; (d) physiological measurements. **Table S8.** List of nuclear proteins obtained after the joint analysis of total protein and nuclear extracts. Spectra were processed as previously described, but one step of *in silico* fractionation was added to remove non-nuclear contaminants. Deflines, E.C. number, and MapMan bins were manually curated. **Table S9.** Correspondence between 454 dataset (Miller *et al.* [33]), Chlamydomonas 5.3, and MapMan analyses. Abundace of 454 detected transcripts is also provided. **Table S10.** Granger causality analyzed by COVAIN (Sun and Weckwerth, 2012 [38]) between metabolites, proteins, and physiological data. Additional file 3: Figure S2. Representation of nitrogen starvation- and recovery-induced changes in photosynthetic pathways using MapMan. Individual plots show the variations in protein abundance at the six studied time points. Only differential proteins (*P* < 0.05) were plotted. Protein abundances were normalized as a percentage of the maximal value in the time series. Additional file 4: Figure S3. Representation of simplified N transport, fixation, and assimilation pathways showing N starvation- and recovery-induced changes using MapMan. Individual plots show the variations in protein abundance at the six studied time points. Only differential proteins (*P* < 0.05) were plotted. Protein abundances were normalized as a percentage of the maximal value in the time series. Additional file 5: Figure S4. Representation of ribosomal proteins using MapMan. Individual plots show the variations in protein abundance at the six studied time points. Only differential proteins (*P* < 0.05) were plotted. Protein abundances were normalized as a percentage of the maximal value in the time series. Additional file 6: Figure S5. Metabolic changes in lipid biosynthetic pathways induced by N starvation. (A) MapMan-based representation of the evolution of the enzymes related to fatty acid biosynthesis and elongation. (B) Variations in the protein abundance of the functional groups involved in lipid metabolism. (C) Evolution of the cellular content of the indicated fatty acids during the time course experiment. (D) sPLS-based network showing the most correlated proteins and metabolites to glycerol and C18:2 nodes. C18:2 was connected to ammonia transport and assimilation proteins, whereas glycerol was mainly connected to glycerol biosynthesis proteins and NADH:ubiquinone oxidoreductases. Interestingly, Cre09.g405500.t1.3 (major lipid droplet protein, with a high importance in the model) links C18:2 and glycerol. This protein was manually annotated to major lipid droplet protein. Other proteins that link both metabolites are a ribosomal protein and g13518.t1 (annotated as amine oxidase). Network interpretation: edge color indicates correlation between nodes, circle diameter indicates the importance of each node within the network, and node color its radiality. Figure was plotted employing Cytoscape 2.8.3. Additional file 7: Figure S6. Representation of nitrogen starvation- and recovery-induced changes in nucleotide metabolism pathways. Individual plots show the variations in protein abundance at the six studied time points. Only differential proteins (*P* < 0.05) were plotted. Protein abundances were normalized as a percentage of the maximal value in the time series. Additional file 8: Figure S7. Classification of the integrated dataset employing different multivariate methods such as principal component analysis, PCA (a,d), partial least squares discriminant analysis, PLS-DA (b,e), and sparse partial least squares, s-PLS (c,f), for which proteins and metebolites were used as predictive variables and physiological measurements as explanatory. For all analyses the first two components allowed an effective classification of the samples based on the sample time. Additional file 9: Figure S8. Simplified sPLS-based network constructed considering only highly correlated nodes (correlation > |0.85|). The three main nodes established different correlations among them, with the glycerol-C18:2 cluster negatively correlated to the others. Interestingly, the fresh weight (FW) cluster is positively linked to the amino acids-myo-inositol cluster by a bridge constituted by the branched amino acids. Val, Leu, and Ile are positively correlated to a monofunctional catalase grouping with amino acids, and on the other side to an acyl-CoA oxidase linked to the FW. Surprisingly one enoyl-ACP-reductase, which is correlated to the amino acids cluster, is negatively correlated to glycerol-C18:2. Glycerol is also negatively correlated to Cre12.g536800.t1.2, a protein showing an uncharacterized domain which is related to FW. Network interpretation: edge color indicates correlation between nodes, circle diameter indicates the importance of each node within the network, and node color its radiality. Figure was plotted employing Cytoscape 2.8.3. Additional file 10: Figure S9. Detailed representation of the fresh weight (FW). FW is correlated to different kinds of enzymes: signaling enzymes, mainly GTPases and Ca2+ dependent, endopeptidases, oxidoreductases, and acyl-CoA synthases, oxidases, and dehydrogenases. Node size is dependent on stress value and node color on radiality. Edge color indicates correlation (red, positive, green negative). Figures were plotted employing Cytoscape 2.8.3. Only highly correlated nodes (correlation > |0.85|) were plotted. Additional file 11: Figure S10. Detailed representation of the amino acid cluster. This cluster showed complex interactions, in which nitrogen assimilation and amino acid metabolism enzymes interact with lipid biosynthesis proteins. Based on this network, some relation between N metabolism and lipid biosynthesis can be established, for example, at the level of one enoyl-ACP-reductase, which correlated to Asp, Arg, Glu, and myo-inositol, or a 3-oxoacyl-ACP reductase linked to Thr and myo-inositol. Thus, myo-inositol is a metabolite that needs to be further investigated since it is in the center of this cluster, linking amino acids, lipids, and sugars. Node size is dependent on stress value and node color on radiality. Edge color indicates correlation (red, positive, green negative). Figures were plotted employing Cytoscape 2.8.3. Only highly correlated nodes (correlation > |0.85|) were plotted.

## Abbreviations

CAH: carbonic anhydrase
CCM: carbon concentrating mechanism
GC-MS: gas chromatography coupled to mass spectrometry
GeLC-LTQ-Orbitrap-MS: gel electrophoresis - liquid cromatography coupled to LTQ-Orbitrap mass spectrometer
KEGG: Kyoto Encyclopedia of Genes and Genomes
LHC: light harvesting complex
MLDP: major lipid droplet protein
PC: principal component
PCA: principal component analysis
sPLS: sparse partial least square
sPLS-DA: sPLS - discriminant analysis
TAG: triacylglycerol
