# Supplementary figures and images for: System-level network analysis of nitrogen starvation and recovery in Chlamydomonas reinhardtii reveals potential new targets for increased lipid accumulation
Source: Biotechnol Biofuels. 2014 Dec 24;7:171. doi: 10.1186/s13068-014-0171-1 (PMC4320484; doi:10.1186/s13068-014-0171-1)

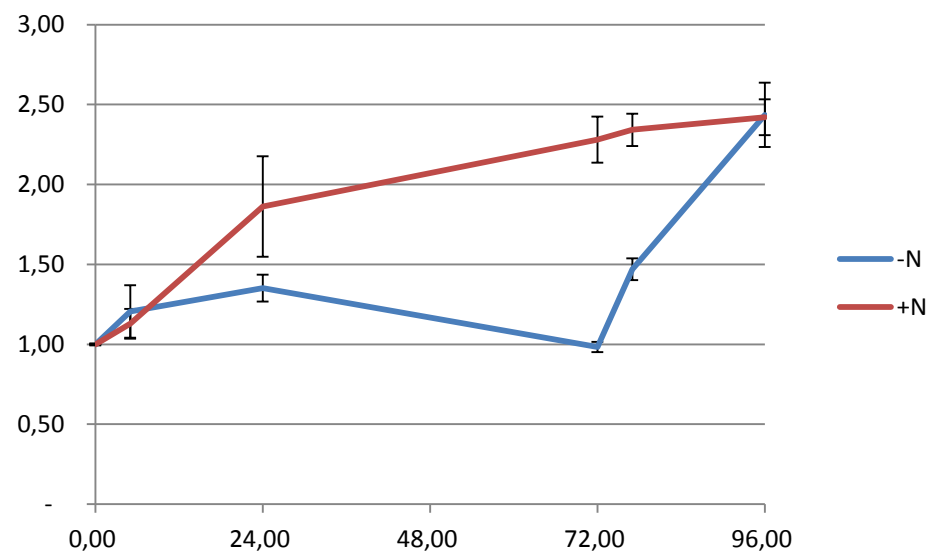

Supplement: Additional file 1: Figure S1. — Comparison of cell growth (fresh weight) of N repleted and depleted cultures of C. reinhardtii CC503 grown under the same conditions employed in this study. Cultures were grown in triplicate. [file 13068_2014_171_MOESM1_ESM.pdf]

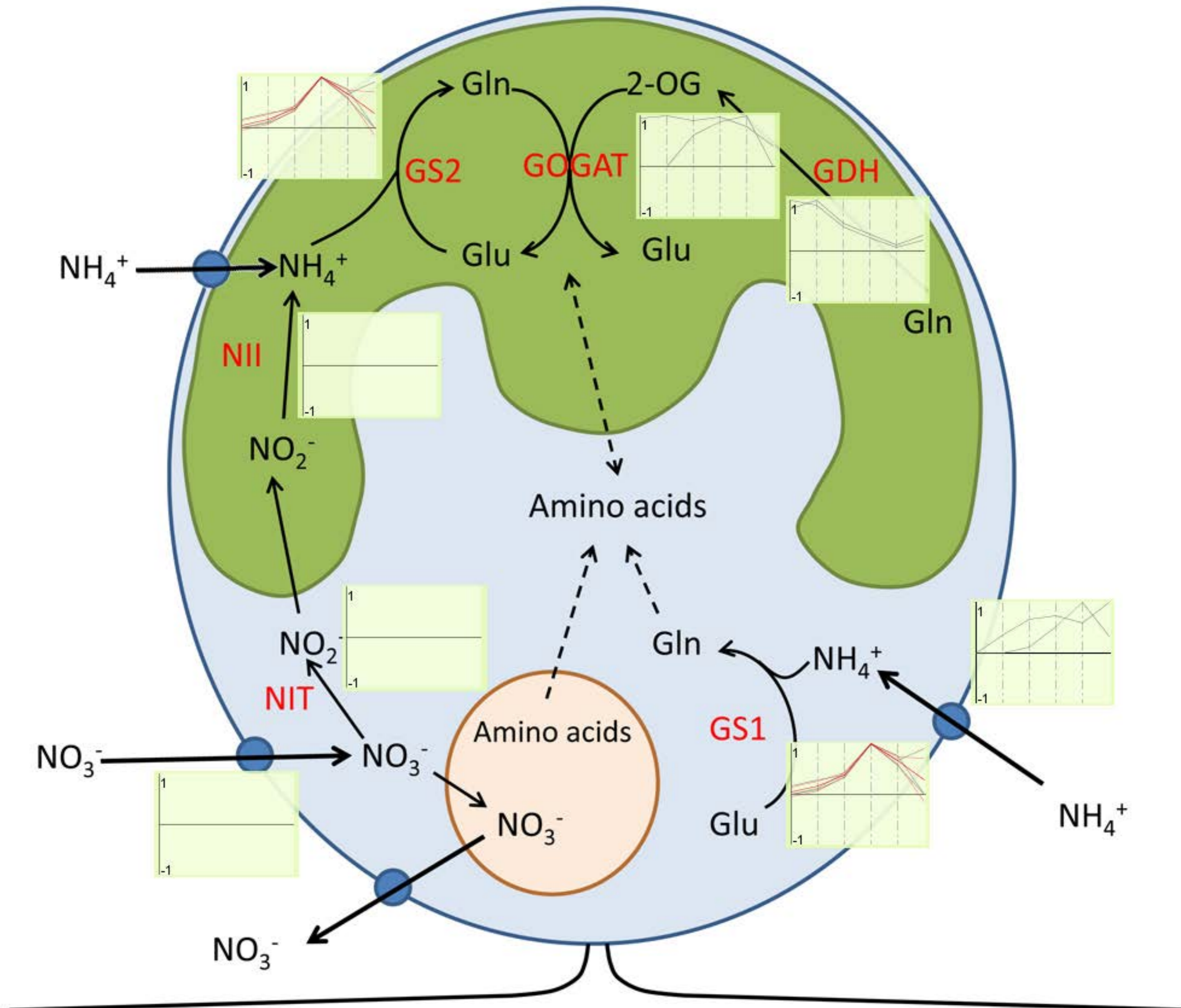

Supplement: Additional file 4: Figure S3. — Representation of simplified N transport, fixation, and assimilation pathways showing N starvation- and recovery-induced changes using MapMan. Individual plots show the variations in protein abundance at the six studied time points. Only differential proteins (P < 0.05) were plotted. Protein abundances were normalized as a percentage of the maximal value in the time series. [file 13068_2014_171_MOESM4_ESM.pdf]

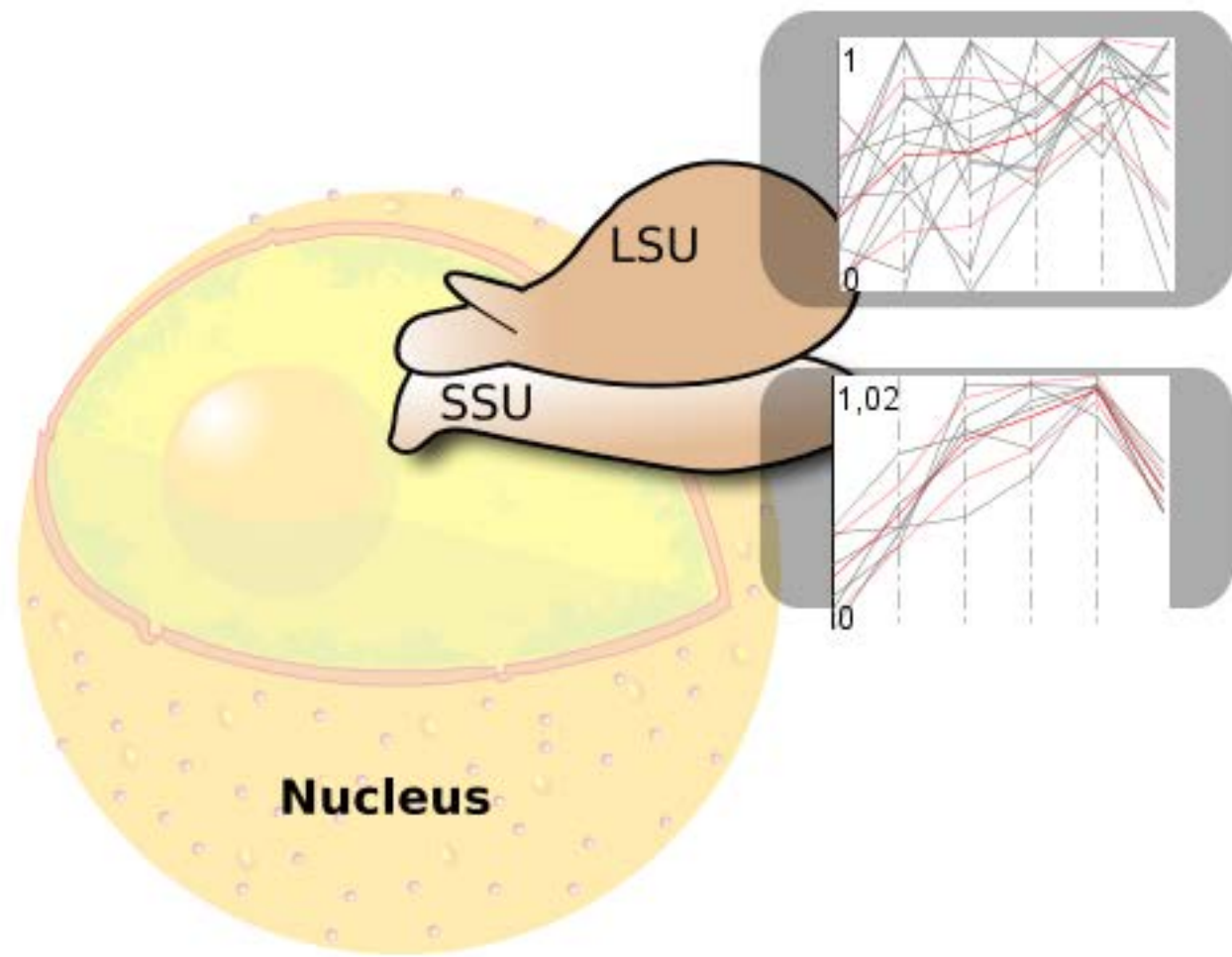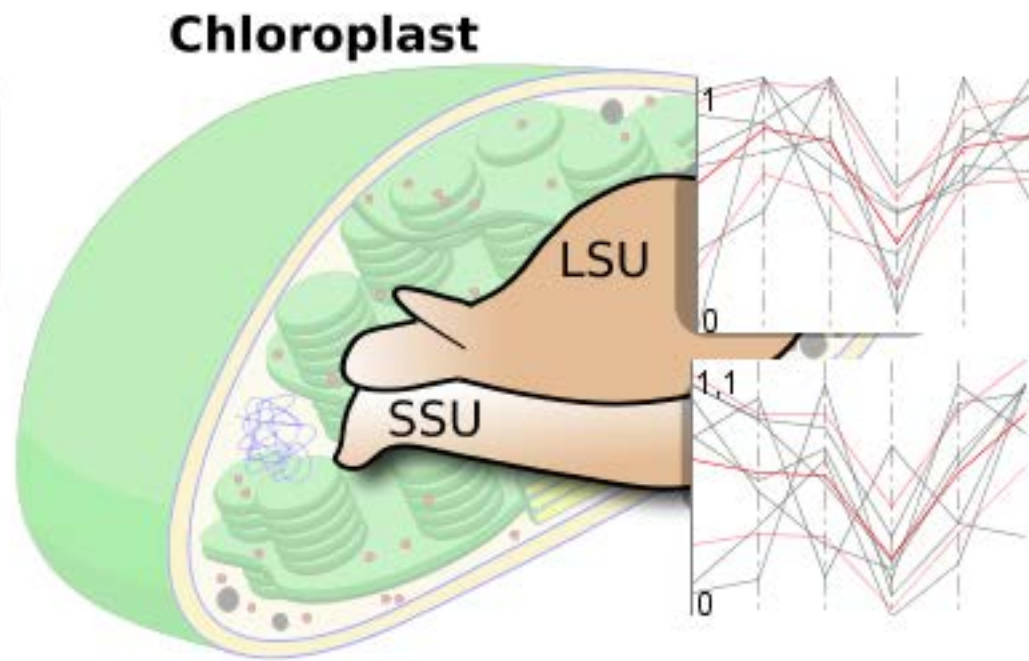

Supplement: Additional file 5: Figure S4. — Representation of ribosomal proteins using MapMan. Individual plots show the variations in protein abundance at the six studied time points. Only differential proteins (P < 0.05) were plotted. Protein abundances were normalized as a percentage of the maximal value in the time series. [file 13068_2014_171_MOESM5_ESM.pdf]

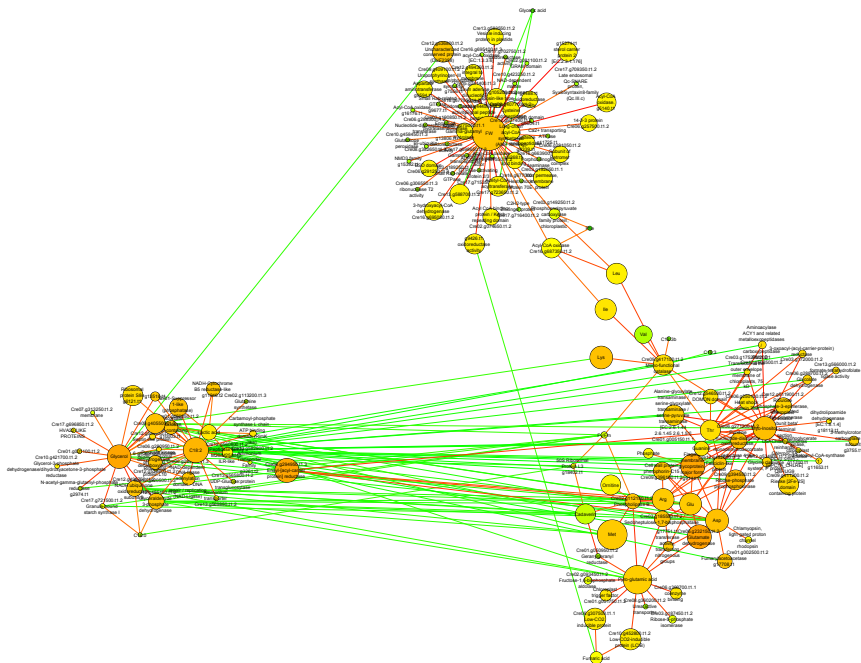

Supplement: Additional file 9: Figure S8. — Simplified sPLS-based network constructed considering only highly correlated nodes (correlation > |0.85|). The three main nodes established different correlations among them, with the glycerol-C18:2 cluster negatively correlated to the others. Interestingly, the fresh weight (FW) cluster is positively linked to the amino acids-myo-inositol cluster by a bridge constituted by the branched amino acids. Val, Leu, and Ile are positively correlated to a monofunctional catalase grouping with amino acids, and on the other side to an acyl-CoA oxidase linked to the FW. Surprisingly one enoyl-ACP-reductase, which is correlated to the amino acids cluster, is negatively correlated to glycerol-C18:2. Glycerol is also negatively correlated to Cre12.g536800.t1.2, a protein showing an uncharacterized domain which is related to FW. Network interpretation: edge color indicates correlation between nodes, circle diameter indicates the importance of each node within the network, and node color its radiality. Figure was plotted employing Cytoscape 2.8.3. [file 13068_2014_171_MOESM9_ESM.pdf]

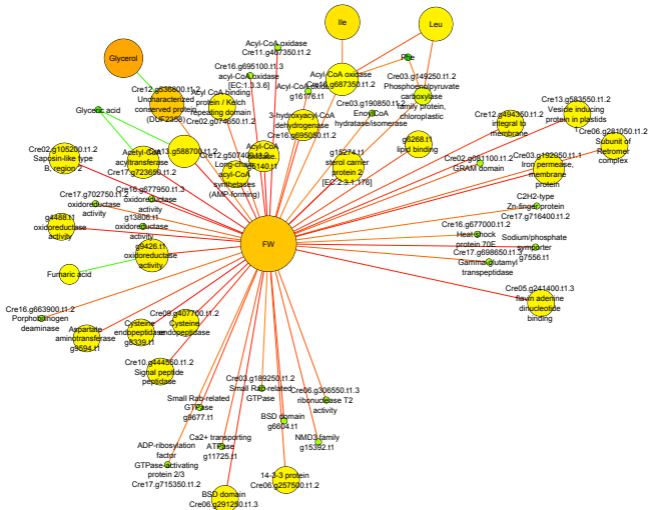

Supplement: Additional file 10: Figure S9. — Detailed representation of the fresh weight (FW). FW is correlated to different kinds of enzymes: signaling enzymes, mainly GTPases and Ca2+ dependent, endopeptidases, oxidoreductases, and acyl-CoA synthases, oxidases, and dehydrogenases. Node size is dependent on stress value and node color on radiality. Edge color indicates correlation (red, positive, green negative). Figures were plotted employing Cytoscape 2.8.3. Only highly correlated nodes (correlation > |0.85|) were plotted. [file 13068_2014_171_MOESM10_ESM.pdf]

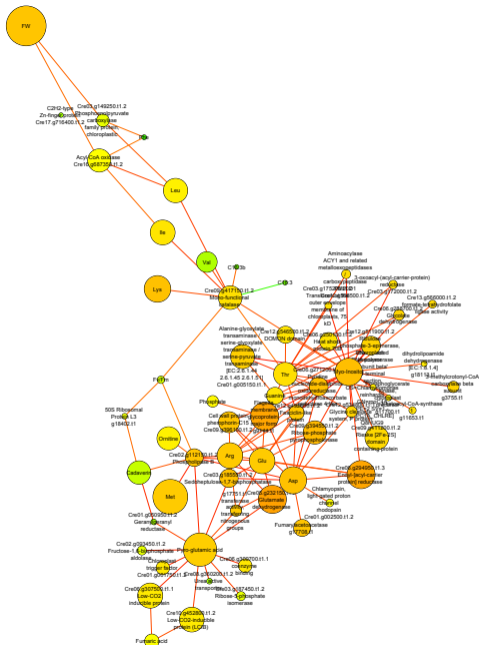

Supplement: Additional file 11: Figure S10. — Detailed representation of the amino acid cluster. This cluster showed complex interactions, in which nitrogen assimilation and amino acid metabolism enzymes interact with lipid biosynthesis proteins. Based on this network, some relation between N metabolism and lipid biosynthesis can be established, for example, at the level of one enoyl-ACP-reductase, which correlated to Asp, Arg, Glu, and myo-inositol, or a 3-oxoacyl-ACP reductase linked to Thr and myo-inositol. Thus, myo-inositol is a metabolite that needs to be further investigated since it is in the center of this cluster, linking amino acids, lipids, and sugars. Node size is dependent on stress value and node color on radiality. Edge color indicates correlation (red, positive, green negative). Figures were plotted employing Cytoscape 2.8.3. Only highly correlated nodes (correlation > |0.85|) were plotted. [file 13068_2014_171_MOESM11_ESM.pdf]
