# Supplementary material for: System-level network analysis of nitrogen starvation and recovery in Chlamydomonas reinhardtii reveals potential new targets for increased lipid accumulation
Source: Biotechnol Biofuels. 2014 Dec 24;7:171. doi: 10.1186/s13068-014-0171-1 (PMC4320484; doi:10.1186/s13068-014-0171-1)

photosynthesis.png
mapping: 20130112 MapManChlamy5+ChloroMito-LV.xls
mapped: 1571 of 1534 data points
visible: 86 data points
data: T0
data: T5
data: T24
data: T72
data: T77
data: T96

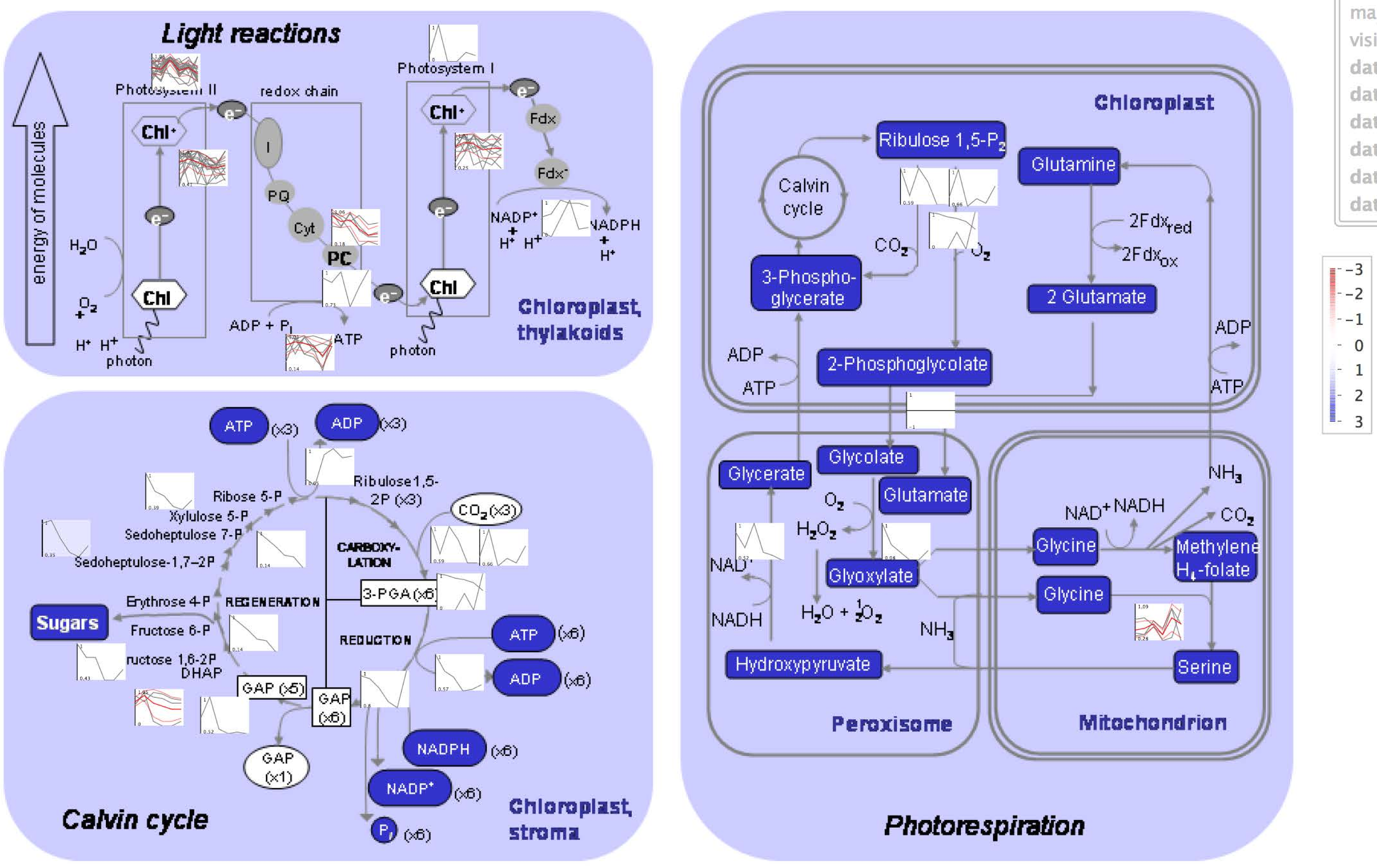

Supplement: Additional file 3: Figure S2. — Representation of nitrogen starvation- and recovery-induced changes in photosynthetic pathways using MapMan. Individual plots show the variations in protein abundance at the six studied time points. Only differential proteins (P < 0.05) were plotted. Protein abundances were normalized as a percentage of the maximal value in the time series. [file 13068_2014_171_MOESM3_ESM.pdf]
