# Supplementary material for: System-level network analysis of nitrogen starvation and recovery in Chlamydomonas reinhardtii reveals potential new targets for increased lipid accumulation
Source: Biotechnol Biofuels. 2014 Dec 24;7:171. doi: 10.1186/s13068-014-0171-1 (PMC4320484; doi:10.1186/s13068-014-0171-1)
Supplement: Additional file 7: Figure S6. — Representation of nitrogen starvation- and recovery-induced changes in nucleotide metabolism pathways. Individual plots show the variations in protein abundance at the six studied time points. Only differential proteins (P < 0.05) were plotted. Protein abundances were normalized as a percentage of the maximal value in the time series. [file 13068_2014_171_MOESM7_ESM.pdf]

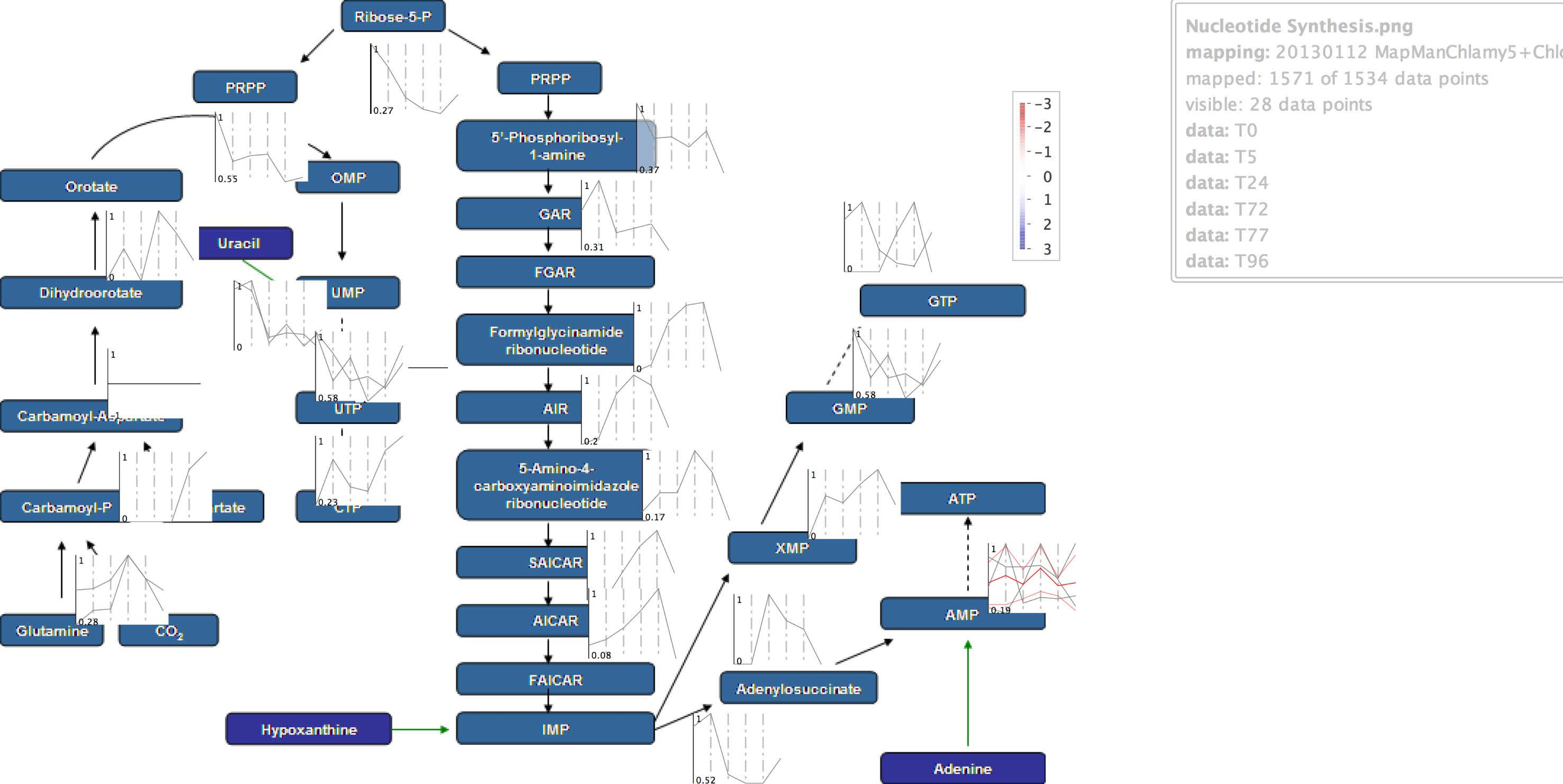
Ribose-5-P
PRPP
PRPP
5'-Phosphoribosyl-1-amine
Orotate
OMP
GAR
Uracil
Dihydroorotate
UMP
FGAR
GTP
Formylglycinamide ribonucleotide
UTP
AIR
GMP
Carbamoyl-P
5-Amino-4-carboxyaminoimidazole ribonucleotide
ATP
XMP
SAICAR
AICAR
AMP
Glutamine
CO_2
FAICAR
Adenylosuccinate
Hypoxanthine
IMP
Adenine
-3
-2
-1
0
1
2
3
Nucleotide Synthesis.png
mapping: 20130112 MapManChlamy5+Chl
mapped: 1571 of 1534 data points
visible: 28 data points
data: T0
data: T5
data: T24
data: T72
data: T77
data: T96
