# Supplementary material for: System-level network analysis of nitrogen starvation and recovery in Chlamydomonas reinhardtii reveals potential new targets for increased lipid accumulation
Source: Biotechnol Biofuels. 2014 Dec 24;7:171. doi: 10.1186/s13068-014-0171-1 (PMC4320484; doi:10.1186/s13068-014-0171-1)
Supplement: Additional file 6: Figure S5. — Metabolic changes in lipid biosynthetic pathways induced by N starvation. (A) MapMan-based representation of the evolution of the enzymes related to fatty acid biosynthesis and elongation. (B) Variations in the protein abundance of the functional groups involved in lipid metabolism. (C) Evolution of the cellular content of the indicated fatty acids during the time course experiment. (D) sPLS-based network showing the most correlated proteins and metabolites to glycerol and C18:2 nodes. C18:2 was connected to ammonia transport and assimilation proteins, whereas glycerol was mainly connected to glycerol biosynthesis proteins and NADH:ubiquinone oxidoreductases. Interestingly, Cre09.g405500.t1.3 (major lipid droplet protein, with a high importance in the model) links C18:2 and glycerol. This protein was manually annotated to major lipid droplet protein. Other proteins that link both metabolites are a ribosomal protein and g13518.t1 (annotated as amine oxidase). Network interpretation: edge color indicates correlation between nodes, circle diameter indicates the importance of each node within the network, and node color its radiality. Figure was plotted employing Cytoscape 2.8.3. [file 13068_2014_171_MOESM6_ESM.pdf]

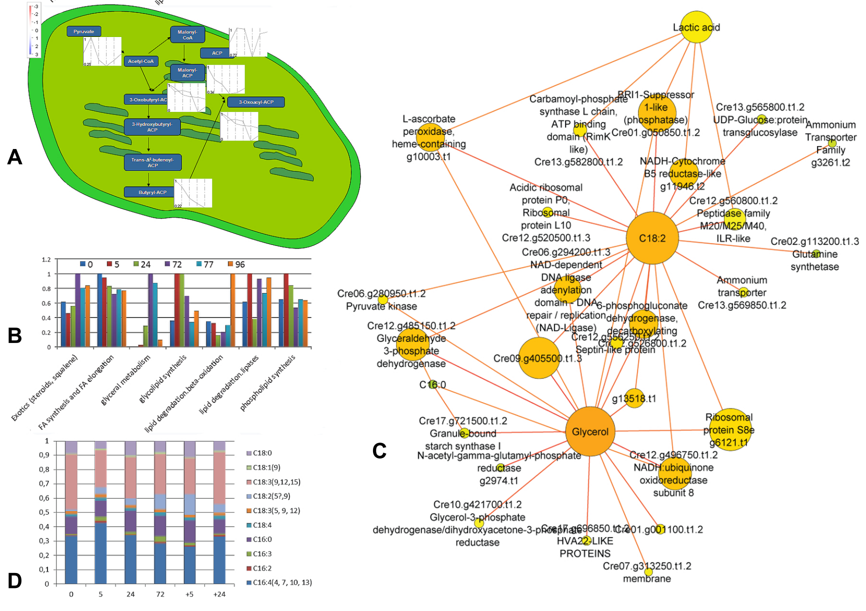
A
Pyruvate
Malonyl-CoA
ACP
Acetyl-CoA
Malonyl-ACP
3-Oxobutyryl-AC
3-Oxoacyl-ACP
3-Hydroxybutyryl-ACP
Trans-Δ2-butenoyl-ACP
Butyryl-ACP
B
0 5 24 72 77 96
Exotics (steroids, squalene)
FA synthesis and FA elongation
glyceral metabolism
glycolipid synthesis
lipid degradation.beta-oxidation
lipid degradation.lipases
phospholipid synthesis
C
Lactic acid
Carbamoyl-phosphate synthase L chain, ATP binding domain (RimK like) Cre13.g582800.t1.2
BRI1-Suppressor 1-like (phosphatase) Cre01.g050850.t1.2
Cre13.g565800.t1.2 UDP-Glucose:protein transglucosylase
Ammonium Transporter Family g3261.t2
L-ascorbate peroxidase, heme-containing g10003.t1
NADH-Cytochrome B5 reductase-like g11946.t2
Acidic ribosomal protein P0, Ribosomal protein L10 Cre12.g520500.t1.3
Cre12.g560800.t1.2 Peptidase family M20/M25/M40, ILR-like
C18:2
Cre02.g113200.t1.3 Glutamine synthetase
Cre06.g294200.t1.3 NAD-dependent DNA ligase adenylation domain - DNA repair / replication (NAD-Ligase)
Ammonium transporter Cre13.g569850.t1.2
Cre06.g280950.t1.2 Pyruvate kinase
6-phosphogluconate dehydrogenase, decarboxylating Cre12.g526800.t1.2
Septin-like protein
Cre12.g485150.t1.2 Glyceraldehyde 3-phosphate dehydrogenase
Cre09.g405500.t1.3
C16:0
g13518.t1
Cre17.g721500.t1.2 Granule-bound starch synthase I
Glycerol
Ribosomal protein S8e g6121.t1
N-acetyl-gamma-glutamyl-phosphate reductase g2974.t1
Cre12.g496750.t1.2 NADH:ubiquinone oxidoreductase subunit 8
Cre10.g421700.t1.2 Glycerol-3-phosphate dehydrogenase/dihydroxyacetone-3-phosphate reductase
Cre17.g696850.t1.2 HVA22-LIKE PROTEINS
Cre01.g001100.t1.2
Cre07.g313250.t1.2 membrane
D
C18:0
C18:1(9)
C18:3(9,12,15)
C18:2(5?,9)
C18:3(5, 9, 12)
C18:4
C16:0
C16:3
C16:2
C16:4(4, 7, 10, 13)
0 5 24 72 +5 +24
