# Supplementary material for: System-level network analysis of nitrogen starvation and recovery in Chlamydomonas reinhardtii reveals potential new targets for increased lipid accumulation
Source: Biotechnol Biofuels. 2014 Dec 24;7:171. doi: 10.1186/s13068-014-0171-1 (PMC4320484; doi:10.1186/s13068-014-0171-1)
Supplement: Additional file 8: Figure S7. — Classification of the integrated dataset employing different multivariate methods such as principal component analysis, PCA (a,d), partial least squares discriminant analysis, PLS-DA (b,e), and sparse partial least squares, s-PLS (c,f), for which proteins and metebolites were used as predictive variables and physiological measurements as explanatory. For all analyses the first two components allowed an effective classification of the samples based on the sample time. [file 13068_2014_171_MOESM8_ESM.pdf]

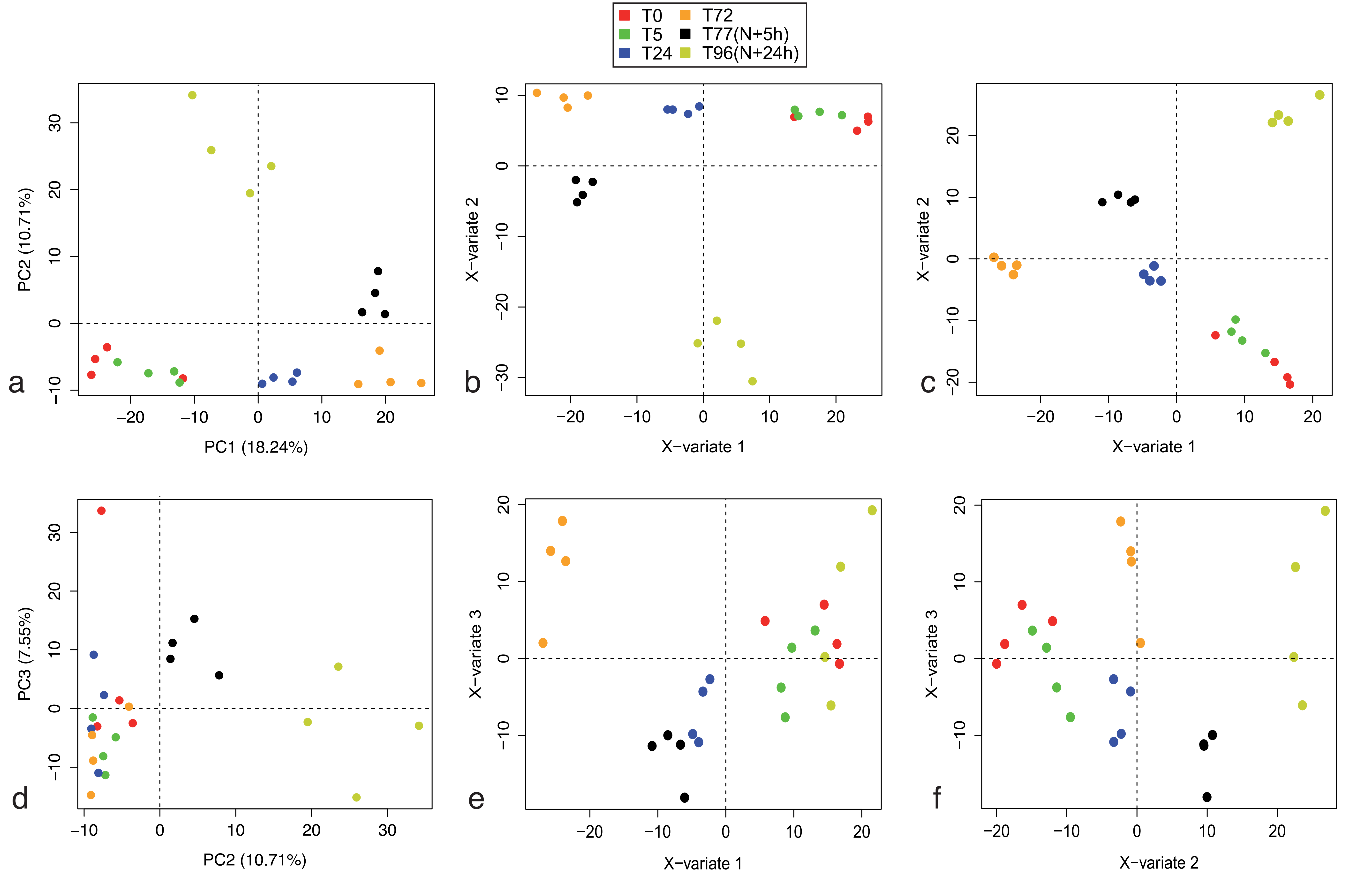

Figure introduction: In the case of PCA (a) glutamate family enzymes (ASS, ASL, OCT, NAG), ammonia metabolism (Cre13.g592200.t1.2), purine biosynthesis proteins (Cre07.g318750.t1.2, Cre08.g364800.t1.2), NADH:ubiquinone oxidoreductase, cGMP-dependent kinases (Cre03.g199050.t1.2), glycolysis enzymes (PK, GAP-DH, PEPC), glyceraldehyde-3P-DH, glycerol, and C18:2 showed high correlations to PC1. Calvin cycle proteins (SBPase, PPE), chloroplastic ATPase, amino acid degradation, polyamine synthase, fatty acid elongation, catalases, and aspartic acid showed a negative correlation to PC1. Fresh weight, alanine, beta oxidation-related proteins (acyl-CoA Oxidases, NADH), oxidoreductases (Cre16.g677950.t1.3, g13806.t1, g4488.t1, g9426.t1), signal peptide and protein peptidases, and tetrapyrrole biosynthesis proteins showed a high correlation to PC2, while organic acids (fumaric and glyceric), phosphate, and photosynthesis-related enzymes (light reaction and carbon fixation) showed a negative correlation. Loading matrix is available in Additional file 2: Table S4. PLS-DA components (b) share most of their variables with PCs, but under this approach Asp, Asn, Glu, calcium signaling (LETM1, Cre06.g263950.t1.3) and hormone metabolism (M20 peptidase family) also showed a significant correlation to first component pointing to differential signaling processes and increased N assimilation pathways. The loading matrix is available in Additional file 2: Table S5. sPLS (c) was intended to classify the samples in the function of the evolution of Fv/Fm, lipids, and FW. Hence, a different set of variables was defined. The first component was strongly and positively correlated to protein degradation (Ser carboxipeptidase, metalloprotease g4429.t1), folding (chaperonin 60B1, HSP70E), and synthesis (ribosomal proteins S1, S3, L3), malic enzyme and SHMT1 involved in photorespiration. On the other hand it showed a negative correlation to mitochondrial ATP synthesis (F1F0 ATP synthase, prohibitin, gamma carbonic anhydrase), cell motility (flagellar associated proteins Cre07.g321400.t1.3, Cre12.g531800.t1.1), glutamine syntases, and amino acid biosynthesis (ASS, OCT, PSAT), organic acid transformation, signaling (GTPase, 12-oxophytodienoate reductase, M20 peptidase Cre17.g728100.t1.2), FAME biosynthesis (GPDH), glycerol, and C16:3. The second component is positively correlated to cell motility (centriole proteome protein, flagellar associated protein Cre02.g081050.t1.2), ammonium transporters, cysteine endopeptidases, signaling (calcium-dependent Cre09.g388850.t1.1, 14-3-3 Cre12.g559250.t1.2), glyoxylate cycle (malate synthase, citrate synthase 2), uracil, and C18:2. Lipid biosynthesis (enoyl-[acyl-carrier protein] reductase, 3-ketoacyl-CoA-synthase 1), cell mating (pherophorin C3 and C15), carbon fixation (CCM Cre06.g307500.t1.1, RBCA, transketolase, sedoheptulose-1,7-bisphosphatase), light reactions (cytochrome b6f), redox homeostasis (Cre06.g271200.t1.2, Cre16.g676150.t1.2), RNA synthesis (Nop56p/Sik1p, DNA-directed RNA polymerase subunit beta, snoRNPs), proteases, and protein targeting enzymes, myo-inositol, serine, and phosphate are negatively correlated to component 2. Loading matrix is available in Additional file 2: Table S6.
